# Co-delivery of antigen and adjuvant by site-specific conjugation to dendritic cell-targeted Fab fragments potentiates T cell responses†

**DOI:** 10.1039/d5cb00014a

**Published:** 2025-05-05

**Authors:** Zacharias Wijfjes, Iván Ramos Tomillero, Camille M. Le Gall, Eric A. W. van Dinther, Frederique Turlings, René Classens, Saikat Manna, Duco van Dalen, Ruud J. R. W. Peters, Kayleigh Schouren, Felix L. Fennemann, Iris M. Hagemans, Floris J. van Dalen, Johan M. S. van der Schoot, Carl G. Figdor, Aaron Esser-Kahn, Ferenc A. Scheeren, Martijn Verdoes

**Affiliations:** a Department of Medical BioSciences, Radboud University Medical Center Nijmegen The Netherlands; b Institute for Chemical Immunology Nijmegen The Netherlands; c IMAGINE! Consortium Nijmegen The Netherlands; d Pritzker School of Molecular Engineering, University of Chicago Chicago USA; e Department of Dermatology, Leiden University Medical Center Leiden The Netherlands m.verdoes1@lumc.nl

## Abstract

The aim of therapeutic cancer vaccines is to induce tumor-specific cellular immune responses. This requires tumor antigens to be efficiently processed and presented by antigen-presenting cells, in particular dendritic cells (DCs). In addition, DCs require maturation to upregulate the surface expression and secretion of T cell costimulatory molecules, which is achieved by co-administration of adjuvants in vaccines. Peptide-based antigen vaccination is an attractive strategy due to the established biocompatibility of peptides as well as the dosing control. To enhance the efficacy of peptide-based vaccines, antigens can be targeted to DCs. Antigen–adjuvant conjugates are known to enhance T cell activation by ensuring DC maturation upon antigen delivery. In this study, we aim to combine these two approaches in a single molecule, and present a DC-targeted antibody fragment–antigen–adjuvant (AAA)-conjugate. We generate the AAA-conjugate through a combination of site-specific sortase-mediated chemoenzymatic ligation and click chemistry. *Ex vivo* T cell activation assays show enhanced efficacy of the AAA-conjugate compared to non-adjuvanted control conjugates. The *in vivo* performance of the AAA-conjugate was suboptimal, which we hypothesize to be a consequence of the hydrophobic character of the conjugate. *In vivo* efficacy was rescued by co-administration of antibody fragment–antigen conjugates and antibody fragment-adjuvant conjugates, in which the antigen and adjuvant were separatedly delivered using two different DC-targeting molecules. In conclusion, this study provides a proof-of-concept for effective *in vivo* antigen-specific T cell activation by targeted delivery of both antigen and adjuvant to DCs in a single or separate molecule using site-specific protein engineering.

## Abstract 2

The aim of therapeutic cancer vaccines is to induce tumor-specific cellular immune responses. In this work, we evaluate molecularly defined DC targeted anti-DEC205 antibody fragment–antigen–adjuvant conjugates (AAA-conjugates) as vaccine platform.

## Graphical abstract

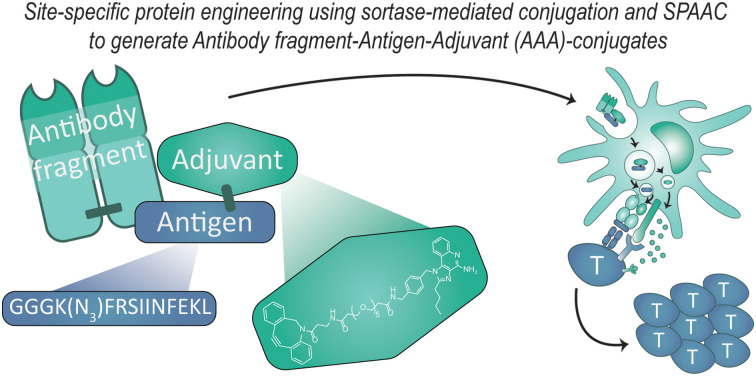

## Introduction

Therapeutic cancer vaccines induce an antigen-specific antitumor response by delivering tumor antigens to antigen-presenting cells (APCs).^1,2^ The tumor vaccine can be nucleic acid- (DNA or mRNA) or protein/peptide-based. Peptide-based vaccines are attractive because of their low cost, high stability and established biocompatibility.^3,4^ In addition, the dosing is better controllable using peptide-based vaccines. This is important, because too high antigen dosing results in recruitment of more low avidity effector T cells, apoptosis of high avidity effector T cells and induces type 1 regulatory T cells.^5–7^ As high avidity effector T cells are more potent than low avidity effector T cells and regulatory T cells dampen immune responses, antigen overdosing results in a weaker immune response.^5,6^ A drawback of peptide-based vaccines is that they are not inherently immunostimulatory and therefore require co-administration of adjuvants. By engaging pattern recognition receptors (PRRs), such as Toll-like receptors (TLRs), adjuvants mature APCs hallmarked by the expression of costimulatory molecules to present antigen-derived epitopes in an immune activating context. This is essential to avoid tolerance towards the antigen.^8^

Adjuvants are often administered systemically in combination with the vaccine. Due to the difference in molecular structure of adjuvant compared to the antigen, different pharmacokinetics results in differences in cell engagement and/or timing. This could lead to pre-activation of APCs before antigen encounter, which suppresses uptake and cross-presentation. The latter is a crucial process for the induction of cytotoxic immune responses in which exogenous antigens are taken up, processed and presented in the context of major histocompatibility complex (MHC) class I.^9^ Also, systemic adjuvant treatment can result in adverse effects such as splenomegaly or T cell recruitment to tissue prior to activation.^10^ Co-encapsulation of antigen and adjuvants in particulate formulations, or antigen–adjuvant conjugation can overcome differences in pharmacokinetics.^11–13^ These strategies have the additional benefit of targeting a single phagosome in APCs, which enhances antigen processing and presentation.^14–16^ Nanoencapsulation is an attractive strategy for antigen–adjuvant delivery as systemic toxicity of the adjuvant can be reduced, yet off-targeting due to particle size is higher compared to antibody-conjugates.^17^ Multiple strategies exist to conjugate adjuvants without compromising the immunostimulant properties.^18^ This facilitates antibody-targeted adjuvant therapy through conjugation of adjuvants to antibodies.^19–21^ Antibody–adjuvant fusions achieve high local concentrations of the adjuvant without the toxicity associated with systemic adjuvant administration.

**Fig. 1:**
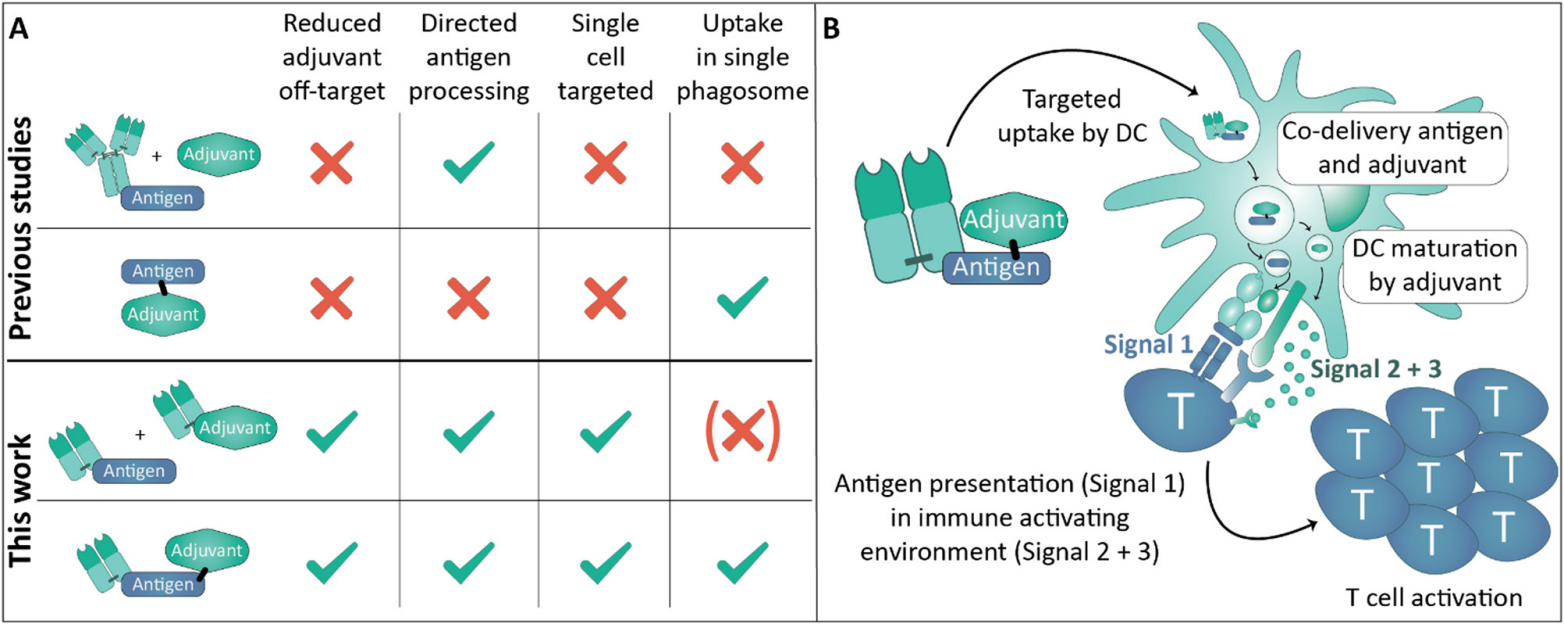
Overview of approaches for targeted antigen and/or adjuvant delivery. (A) Overview of various combinations of (un)targeted (co-)delivery of antigen and adjuvant to dendritic cells and their benefits and drawbacks. Targeted antigen–adjuvant conjugates combine the benefits of targeted antigen delivery and antigen–adjuvant conjugates. (B) Mechanism of activation for antibody fragment–antigen–adjuvant (AAA)-conjugate presented in this study. The conjugate is taken up *via* DEC205 by dendritic cells, which are activated by the adjuvant (TLR7/8-agonist) to present the OT-I antigen (Signal 1) in an immunostimulatory fashion characterized by receptor co-stimulation (Signal 2) and an immunostimulatory cytokine secretion (Signal 3). The dendritic cell interacts with antigen-specific T cells to induce a cytotoxic antitumor immune response. In the case of separate antigen and adjuvant delivery, the mechanism for activation is similar, yet adjuvant and antigen could be delivered to distinct phagosomes.

Targeted delivery of antigens to APCs such as dendritic cells (DCs) is established to improve T cell responses towards the delivered antigen.^22,23^ Targeting specific DC receptors enables control of the type of immune response induced, to for example favor the cytotoxic immune responses required for killing of tumor cells.^24^ Most antibody conjugates used as cancer vaccine target antigen to DCs and co-administer adjuvant systemically. Examples of single molecule DC-targeted antibody-based antigen–adjuvant co-delivery systems are scarce and utilize protein-based adjuvants in fusion proteins or random conjugation of TLR agonists.^25–27^ Protein-based adjuvants are typically less potent than non-proteinaceous agonists, and random conjugation strategies yield heterogeneous products with varying payload-to-drug ratios and the risk of compromised target recognition by conjugation in the binding region of the antibody.^28–30^ Site-specific conjugation of potent adjuvants such as TLR agonists overcomes these drawbacks, as exact control of conjugation site and amount of conjugated payload are achieved. We set-out to combine the benefits of targeted antigen delivery and antigen–adjuvant fusion in a single site-specific generated antibody-based conjugate (Fig. 1).

In this work, we present molecularly defined DC-targeted anti-DEC205 AAA-conjugates. We describe their generation *via* site-specific conjugation using a combination of sortase-mediated chemoenzymatic ligation and strain-promoted azide–alkyne click (SPAAC) chemistry. We opted for sortase-mediated conjugation due to the site-specific character of the technique, as well as the synthetic availability of the substrate, a triple glycine motif, by solid-phase peptide synthesis (SPPS). Additionally, this allows for facile incorporation of an azide-handle, which can be used in the SPAAC reaction to generate a molecularly defined conjugate. Then, we fully characterize the AAA-conjugates and assess T cell activation induced by our conjugates *ex vivo* and *in vivo*.

## Experimental

### Ethical statement

All animal studies were approved by the local authority for the Ethical Evaluation of Animal Experiments and Animal Welfare (Instantie voor Dierenwelzijn Radboudumc). Mice were kept in accordance with federal and state policies on animal research and Annex III of the EU Directive (Directive 2010-63-EU).

### Cell lines culture conditions

NLDC-145 hybridoma cells were cultivated in a 5% CO_2_ humidified incubator at 37 °C in complete RPMI medium (RPMI1640 (ThermoFisher, Gibco™, 11875093) supplemented with 10% heat-inactivated fetal bovine serum (HyClone, SV35959), 4 mM stable glutamine (Capricorn Scientific, STA-B), 1× antibiotic–antimycotic (ThermoFisher, Gibco™, 15240062) and 50 μM 2-mercaptoethanol (Sigma-Aldrich, M6250-100 mL)) in T75 flasks (VWR, Greiner Bio-One, 658170). Both floating and adherent population were passaged 1 : 10 upon reaching >90% confluency using a cell scraper to loosen the adherent population.

JAWS II dendritic cells were cultivated in a 5% CO_2_ humidified incubator at 37 °C in MEM α, nucleosides medium (ThermoFisher, Gibco™, 12571063) supplemented with 20% heat-inactivated fetal bovine serum (HyClone, SV35959), 4 mM stable glutamine (Capricorn Scientific, STA-B), 1× non-essential amino acids (ThermoFisher, Gibco™, 11140050), 1× antibiotic–antimycotic (ThermoFisher, Gibco™, 15240062), 0.75% sodium carbonate (ThermoFisher, Gibco™, 25080094), 1 mM sodium pyruvate (ThermoFisher, Gibco™, 11360070), 50 μM 2-mercaptoethanol (Sigma-Aldrich, M6250-100 mL) and 10 ng mL^−1^ recombinant mouse GM-CSF (Peprotech, 315-03) in T75 flasks (VWR, Greiner Bio-One, 658170). Both floating and adherent population were passaged 1 : 4 upon reaching >80% confluency using 0.25% trypsin–0.03% EDTA to loosen the adherent population.

### CRISPR/HDR genome editing

NLDC-145 hybridoma were genetically modified using CRISPR/HDR-technology as described.^31^ In short, cells were transfected with HDR-templates encoding Fab fragments equipped with a 4S9-sortase recognition motif and a His-tag and subsequently cultivated under blasticidin selection pressure. Upon reaching confluency, single cell clones were made. These clones were screened using dot blotting, western blotting and FACS for high production of the desired Fab fragments. The highest producing clone was selected and used for production of the Fab fragments.

### Production and isolation of Fab fragments

Genetically modified NLDC-145 hybridomas were cultivated in a Corning® CELLine Disposable Bioreactor (Corning, 353137) according to manufacturer's protocol for 6–12 weeks. Supernatant was collected every 10 days, filtered through a 0.2 μm filter (ThermoFisher, 564-0020) and stored at −20 °C. For Fab fragments isolation, all fractions of stored supernatant were thawed, pooled and supplemented with 10 mM imidazole. 5 mL of HisPur™ Ni-NTA resin (ThermoFisher, 88221) was washed with PBS and added to the supernatant. After incubation for 2 h at 4 °C, the resin was divided over two disposable columns and washed with 500 mL 10 mM imidazole in PBS. The Fab fragments were eluted from the resin with 10 mL 250 mM imidazole in PBS. Buffer exchange to PBS was performed using Amicon® Ultra Centrifugal filter, 10 kDa MWCO (Millipore, UFC9010) according to manufacturer's protocol. Yields were determined using a Nanodrop™ 2000 spectrophotometer (ThermoFisher) and purity was assessed by reducing SDS-PAGE (12%) analysis.

### Peptide synthesis

25 mL polypropylene syringes with a porous disc were used for solid-phase peptide synthesis. For short incubation (<5 min), the reactions were manually stirred with a Teflon stick, and for longer incubation times the reactions were stirred on a Unimax 1010 shaker. In between cycles, solvents were removed by vacuum filtration. All the reactions were carried out at room temperature (*ca.* 21 °C). Wang resin with pre-loaded amino acids was used for peptide synthesis. Peptide coupling was performed by adding a 5 min pre-activated solution of Fmoc-amino acid-OH, DIPCDI and Oxyma Pure (3 eq. each respect to resin loading) into the free amino-peptidyl resin. Each coupling was carried out at rt for 40 min with constant shaking. Kaiser or chloranil test for N-terminal primary amines or proline derivatives respectively, were performed after each coupling to evaluate coupling completion. Re-couplings were performed in case of a positive Kaiser or chloranil test. After cleavage, peptides were precipitated in cold Et_2_O by ultracentrifugation using an Allegra™ 21 R (Beckman Coulter) centrifuge and purified on Preparative-HPLC-ESI system (Waters) using C18 reverse phase column. Peptide masses were initially predicted by ChemDraw Professional 15.0 (PerkinElmer, Massachusetts, USA) and compared to acquired masses after LC-MS measurements.

GGGK(N3)FRSIINFEKL: isolated 198 mg, analysis C18 column, gradient from 95 : 5 to 0 : 100 H_2_O/MeCN in 37 min at rt, purity 98.7%. Calculated mass: C_72_H_114_N_22_O_19_ = 1591.84 g mol^−1^. Mass observed: 1592.52, 796.64, 531.32 corresponding to M + H^+^, (M + 2H^+^)/2 and (M + 3H^+^)/3 respectively.

### Sortase-mediated chemoenzymatic ligation and SPAAC

Anti-mDEC205 Fab fragment (1 eq., 20 nmol, 1 mg), sortase 4S9 (0.25 eq., 5 nmol, 0.09 mg) and GGGK(N_3_)FRSIINFEKL peptide (40 eq., 800 nmol, 1.16 mg) were added in sortase buffer (50 mM Tris–HCl, 150 mM NaCl, 2 mM CaCl_2_, pH = 7.5, 10% v/v DMSO) and incubated for 2 h at 37 °C. Then, 100 μL of HisPur™ Ni-NTA resin (ThermoFisher, 88221) was added and incubated for 15 min at rt to remove remaining starting material and sortase. The solution was centrifuged (10 000 rcf, 1 min) and the clear supernatant was loaded onto a size exclusion NGC Chromatography System (Bio-Rad). The intermediate product was isolated using a flowrate of 0.3 mL min^−1^ PBS and directly used in a SPAAC. For this, DBCO-functionalized adjuvant (5 eq., 100 nmol, 0.08 mg) was added and the reaction was incubated for 16 h at rt. The final product was isolated by size exclusion NGC Chromatography System (Bio-Rad) using a flowrate of 0.3 mL min^−1^ PBS. Yields were determined using a Nanodrop™ 2000 spectrophotometer (ThermoFisher) and purity was assessed by reducing SDS-PAGE (12%) analysis and MALDI-TOF. Endotoxin removal was performed as described below.

### Endotoxin removal and analysis

Endotoxins were removed using a Pierce endotoxin removal kit (88274, ThermoFisher) according to the manufacturer's protocol. In short, Pierce endotoxin removal column was prepared by removing storage buffer, washing overnight with 0.2 M sodium hydroxide, washing with 2 M sodium chloride, washing with endotoxin-free MilliQ water, and washing three times with PBS. The samples were applied to a dry column and incubated for 2 h at room temperature. The samples were collected by centrifugation and endotoxin levels were analyzed by chromogenic Limulus amebocyte lysate test performed by the Radboudumc Nijmegen pharmacy department.

### Binding assay

DEC205-expressing JAWSII were seeded at 30 000 cells in a V-bottom plate. Conjugates were added in a 1 : 3 dilution series and incubated for 20 min at 4 °C. Subsequently, commercially available anti-DEC205-PE (Biolegend, 138214, 1 : 1000 dilution) was added and incubated for 20 min at 4 °C. Cells were prepared for FACS analysis as described below and analyzed using a FACSVerse™ (BD Biosciences) for PE-signal.

### Serum stability assay

The AAA-conjugate was incubated at 37 °C in 50% mouse serum (Invitrogen) in PBS for timepoints mentioned in the figure. Afterwards, a binding assay as described above was performed.

### *Ex vivo* bone-marrow dendritic cell activation assay

Flt3L BMDCs were generated as described below and seeded in 25 μL complete RPMI at 100 000 cells per condition in a U-bottom plate. Adjuvants were provided at 1 μM or 1 μg mL^−1^ in 25 μL PBS and the cells were incubated overnight in a 5% CO_2_ humidified incubator at 37 °C. The following day, supernatant was collected for ELISA analysis and cells were prepared for FACS analysis using a FACSVerse™ (BD Biosciences).

### *Ex vivo* OT-I cell activation assay

Flt3L BMDCs were generated as described below and seeded in 25 μL complete RPMI at 20 000 cells per condition in a U-bottom plate. Vaccine conjugates were added to the BMDCs and incubated for 2 h at 37 °C in a final volume of 50 μL. Cells were washed with PBS and 100 000 CellTrace™ violet-stained OT-I cells were added. The BMDC-OT-I co-culture was incubated for 72 h at 37 °C in 200 μL complete RPMI medium. After 72 h, supernatant was collected for ELISA analysis and cells were prepared for FACS analysis using a FACSLyric™ (BD Biosciences).

### Flt3L bone-marrow dendritic cell generation

Hindlegs of 6–8 weeks old female C57BL/6J mice (Charles River) were dissected. Tibia and femur were isolated and cut open with a scalpel on the edge of the bones. Bone-marrow was flushed out with PBS and collected. Ammonium-chloride-potassium (ACK) lysis was performed, and bone-marrow cells were seeded per 15–25 × 10^6^ cells in a T75 flaks in complete RPMI medium supplemented with 200 ng mL^−1^ hFlt3-ligand (Miltenyi Biotec, 130-096-479) and 50 μM fresh β-mercaptoethanol. After 8 days, bone-marrow dendritic cells (BMDCs) were harvested and resuspended in complete RPMI medium for experiments.

### OT-I cell isolation

Transgenic 6–8 weeks old female OT-I mice (C57BL/6-Tg(TcraTcrb)1100Mjb/Crl, Charles River) were killed by cervical dislocation. Spleen and inguinal lymph nodes were harvested and meshed on separate 100 μm cell strainers (Corning, 431752). Lymphocytes were kept on ice, while splenocytes were subjected to ACK lysis. After lysis, splenocytes were pooled with the lymphocytes and CD8^+^ OT-I cells were isolated using magnetic-assisted cell sorting according to manufacturer's protocol (CD8α T Cell Isolation Kit, mouse, Miltenyi Biotec). OT-I cells were stained with CellTrace™ Violet (ThermoFisher) for 20 min at 37 °C, recovered in complete medium, washed and resuspended in complete RPMI medium for experiments.

### *In vivo* OT-I cell activation assay

CellTrace™ violet-stained OT-I cells were obtained as described below. 6–8 weeks old female C57BL/6J were injected intravenously with 1 × 10^6^ OT-I cells in 100 μL PBS. For the subcutaneous injections, mice were injected the following day with two subcutaneous injections of 20 pmol vaccine constructs (±1 μg, ±50 ng g^−1^) in 100 μL on the left flank and on the right flank of the mice. Two days after, mice were killed and inguinal lymph nodes and spleens were harvested. Cells were prepared for FACS analysis and analyzed using a FACSLyric™ (BD Biosciences). For the intravenous injections, mice injected intravenously the following day with 20 pmol vaccine constructs (±1 μg, ±50 ng g^−1^) in 100 μL. Three days after, mice were killed and inguinal lymph nodes and spleens were harvested. Cells were prepared for FACS analysis and analyzed using a FACSLyric™ (BD Biosciences).

### RP-HPLC analysis

The conjugates were diluted in PBS to 0.2–0.4 mg mL^−1^ before acquisition on a Vanquish flex UHPLC system (ThermoFisher) equipped with a MAbPac™ (4 μM, 2.1 × 100 MM) Reversed Phase HPLC column (088647, ThermoFisher) using a mobile phase consisting of a 30–60% gradient of acetonitrile in water + 0.1% trifluoroacetic acid, 12 min with a flow of 0.550 mL min^−1^ with detection at 215 nm and 280 nm. The data was analyzed using Chromeleon™ Chromatography Data System (CDS) Software (Thermo Scientific™).

### SDS-PAGE analysis

12% SDS-PAGE gels with a 2.25% stacking layer were prepared manually. Samples were diluted in Laemmli's 4× sample buffer (40% glycerol, Tris/HCl (0.2 M, pH 6.8), 8% SDS, 10% BME, and 0.04% bromophenol blue) and denatured at 95 °C for 5 min. Subsequently, samples were loaded at 0.5 μg protein and the gel was run at 70 V for 15 min, followed by 130 V for 90 min. Proteins were stained by SYPRO™ Ruby stain (S12000, ThermoFisher) according to manufacturer's protocol and visualized using an Amersham Typhoon 5, gel and blot imaging system (Cytiva).

### MALDI-TOF analysis

MALDI-TOF samples were prepared by applying 0.5 μL of sinapinic acid (*trans*-3,5-dimethoxy-4-hydroxycinnamic acid, D7927, Sigma Aldrich) matrix in 1 : 1 v/v MilliQ : ACN on a MALDI plate, followed by 0.5 μL 0.3–1 mg mL^−1^ sample and again 0.5 μL sinapinic acid matrix. Samples were acquired on a Microflex® LRF MALDI-TOF system (Bruker) and analyzed using mMass software.^32^

### ELISA analysis

Supernatant was collected, stored at −20 °C and thawed for ELISA analysis. Manufacturer's protocol was followed for mIL-2 (Invitrogen IL-2 Mouse Uncoated ELISA kit, 88-7024-88, ThermoFisher), mIFNγ (Invitrogen IFN gamma Mouse Uncoated ELISA kit, 88-7314-88, ThermoFisher) and mTNFα (Invitrogen mouse TNF alpha Uncoated ELISA Kit, 88-7324-88, ThermoFisher). In short, high affinity plates were coated with capture antibody overnight at 4 °C and washed with wash buffer (PBS supplemented with 0.05% TWEEN® 20 (P9416, SigmaAldrich)). Samples were diluted (1 : 20 IL-2, 1 : 100 IFNγ, 1 : 5 TNFα), applied on the ELISA plate and incubated overnight at 4 °C. The following day, signal was visualized by applying detection antibody, avidin-HRP, TMB solution and 2 M sulfuric acid with incubation steps and washing in between. The samples were analyzed at wavelength 450 nm for signal and 570 nm for background subtraction using an iMark microplate reader (1681130, Bio-Rad). Analysis was performed with Prism8 (Graphpad) using sigmoidal 4PL modeling.

### Flow cytometry analysis

For FACS analysis, cells were washed with PBS and stained for 20 min at room temperature with 50 μL life/death staining eBioscience™ Fixable Viability Dye eFluor™ 780 (1 : 2000 dilution, 47-4317-82, ThermoFisher). Subsequently, cells were washed once with PBS and antibody mixes (described below) were added for 30 min at 4 °C. Cells were washed twice with PBA, taken up in 100 μL PBA and FACS analyses were performed on a FACSLyric™ (BD Biosciences) or a FACSVerse™ (BD Biosciences).

### Antibodies

The following anti-mouse antibodies have been used throughout the studies presented in this manuscript; DEC205-PE (NLDC-145, 1 : 1000, 138214, Biolegend), CD8-PerCP (53-6.7, 1 : 100, 100701, Biolegend), CD25-FITC (PC61, 1 : 100, 102005, Biolegend), CD137-APC (17B5, 1 : 100, 17-1371-82, ThermoFisher), CD11c-APC (N418, 1 : 200, 117309, Biolegend), CD40-PerCP/Cy5.5 (3/23, 1 : 100, 124624, Biolegend), CD80-AF488 (16-10A1, 1 : 100, 104715, Biolegend), I-A/I-E-BV510 (M5/114.15.2, 1 : 100, 107635, Biolegend).

## Results and discussion

**Fig. 2:**
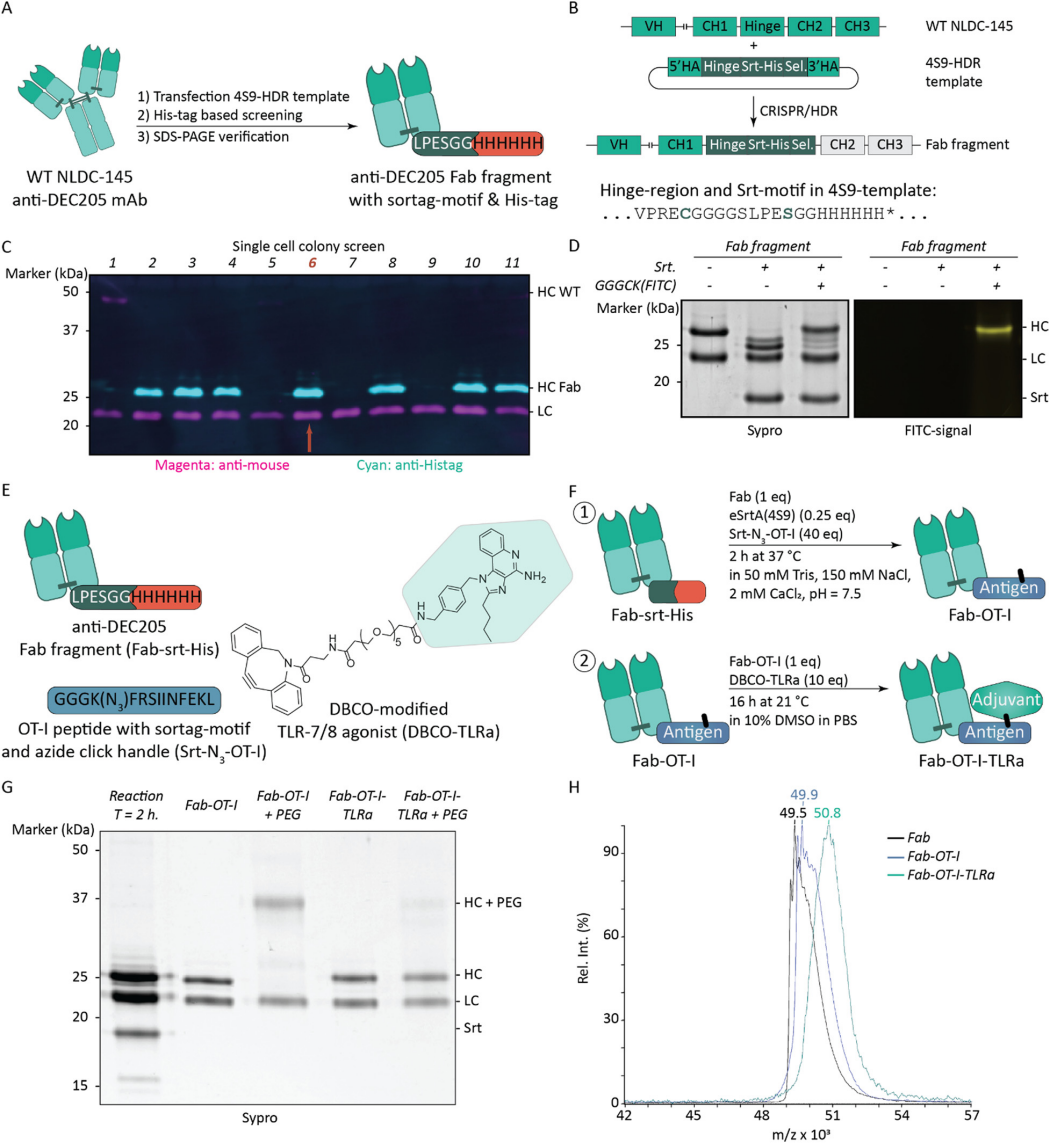
Generation of molecularly defined antibody fragment–antigen–adjuvant conjugates. (A) Schematic overview of workflow to obtain aDEC205 (NLDC-145) Fab fragments equipped with a eSrt4S9 sortase-motif and His-tag. (B) The 4S9-HDR template contains an additional cysteine in the hinge-region compared to our first generation of Fab fragments 31 to improve stability, and an eSrt4S9-motif for more facile chemoenzymatic ligation. (C) Western blot analysis indicates incorporation of the HDR-template in single cell colony 2, 3, 4, 6, 8, 10 and 11 as a positive signal for the His-tag is observed at the expected height of ±25 kDa. Colony 6 was selected for production. (D) Fluorescent and Sypro Ruby stained reducing SDS-PAGE (12%) analysis of sortase-mediated transpeptidation of GGGCK(FITC) to Fab fragment. (E) Overview of substrates used to obtain the AAA-conjugate. Peptide sequences are depicted for the sortase recognition-motif and His-tag attached to the Fab fragment (Fab-srt-His), and OT-I peptide with e4S9 sortase recognition-motif and azide click-handle (Srt-N3-OT-I). The chemical structure of the DBCO-modified TLR-7/8 agonist (DBCO-TLRa) is depicted and its synthesis is described in the supplemental methods. (F) Schematic overview of reaction conditions to obtain AAA-conjugate. (G) Reducing SDS-PAGE (12%) analysis of AAA-conjugate (Fab-OT-I-TLRa). Analytical click reaction with DBCO-PEG5k demonstrates availability azide in HC as depicted by a mass shift in lane 3 corresponding to attachment of the PEG5k, whereas in lane 5 no HC mass shift is observed as the azide is consumed by the DBCO-adjuvant. (H) MALDI-TOF analysis of Fab fragments. Mass shifts correspond to removal of His-tag and attachment of antigen and loss of His-Tag, and attachment of adjuvant respectively.

To obtain molecularly defined AAA-conjugates, we engineered anti-mouse DEC205 (CD205, Clec13b) Fab fragments C-terminally functionalized with a sortase recognition-motif for chemoenzymatic modification followed by a polyhistidine (His)-tag for affinity-based purification (Fab-srt-His). DEC205 is a C-type lectin mainly expressed on CD8^+^ DCs in mouse and is a well-characterized target for cancer vaccines to enhance cross-presentation.^22,33^ While Fab fragments can be obtained using transient recombinant expression methods, we opted for genetically engineering of NLDC-145 hybridomas using our established CRISPR/HDR-methodology to obtain a stable cell line producing anti-DEC205 Fab-srt-His (Fig. 2A).^31^ We improved our previously published Fab fragment by ensuring disulfide bridge formation between the heavy chain (HC) and the light chain (LC) to increase the stability of the Fab fragment (Fig. 2B and Table S1, ESI†), and by exchanging the eSrt3M-motif (LPETGG) for an eSrt4S9-motif (LPESGG) for more facile ligation.^34^ eSrt4S9 is an improved sortase that results in less hydrolytic byproducts compared to eSrt3M.^35^ After transfecting NLDC-145 hybridomas, we performed limiting dilution to obtain monoclonal cell cultures. Subsequent western blot analysis of the supernatants revealed that multiple clones were successfully genetically modified and produced His-tag functionalized truncated HC (Fig. 2C). One clone (clone #6) was selected for production and the desired Fab fragment was isolated in high yield (70 mg) (Fig. S1, ESI†). To validate structural integrity of the sortase recognition-motif, we attempted the attachment of a FITC-labeled peptide (GGGCK(FITC)) through sortase-mediated ligation. Fluorescent SDS-PAGE analysis (Fig. 2D) revealed a fluorescent signal on the Fab fragment's HC with a molecular weight similar to the starting material, while minimal formation of lower molecular weight hydrolysis product was observed. This indicates that the Fab fragments can be efficiently modified site-specifically.

### Preparation of AAA-conjugate

With Fab-srt-His in hand, we set-out to prepare the AAA-conjugate (Fig. 2E and F). To achieve this, we synthesized the peptide GGGK(N_3_)FRSIINFEKL, which contains a triglycine-motif for sortase-mediated ligation, an azido-lysine as click-handle, an FR-dipeptide cleavage-motif, and the epitope SIINFEKL. The latter is an extensively used dominant CD8-epitope derived from the model antigen ovalbumin (OVA).^36^ The FR-dipeptide motif has been shown to improve proteasomal processing resulting in enhanced cross-presentation of the antigenic epitope in the context of antibody-based DEC205-targeting.^37^ Sortase-mediated conjugation was performed and the product was analyzed by reducing SDS-PAGE analysis (Fig. 2G). A small mass shift of the HC of the Fab fragment was observed, indicating attachment of the click-handle and antigen containing peptide (Fig. 2G, lane 2). To further validate formation of the azide-containing intermediate, an analytical SPAAC reaction with DBCO-PEG_5k_ was performed, which resulted in a near-quantitative molecular weight shift on SDS-PAGE of the HC (Fig. 2G, lane 3). In parallel, conjugation of DBCO-modified fluorophores to the intermediate demonstrated the availability of the azido-lysine for SPAAC and provided fluorescently labeled products (Fig. S2, ESI†). Next, a DBCO-modified TLR agonist (DBCO-TLRa, Fig. 2E) was attached to the intermediate using SPAAC. We opted for an imiquimod-derivative with a SPAAC-handle, which design was based on the benzylic imidazoquinoline backbone reported by Shukla and co-workers.^38^ TLR-7 and TLR-8 are located in endosomes and are known to efficiently mature DCs upon ligand engagement, which makes these receptors attractive targets for our AAA approach.^39^ SPAAC with DBCO-TLRa resulted in formation of the AAA-conjugate, as a quantitative mass shift of the intermediate was observed (Fig. 2G, lane 4). Again, an analytical click reaction with DBCO-PEG_5k_ was performed and this time no shift of the HC was observed, indicating full conversion of the intermediate to the final product (Fig. 2G, lane 5). Matrix assisted laser desorption/ionization time-of-flight (MALDI-TOF) analysis confirmed product formation and mass shifts were observed corresponding to the addition of the antigen and adjuvant respectively (Fig. 2H). Non-targeted controls were prepared similarly using a Fab fragment targeting human CD20 (Fig. S3, ESI†). Finally, we tested for endotoxins in our vaccines and, if necessary, removed them to ensure that the adjuvant effects observed in functional experiments could be attributed specifically to the conjugated TLR-7/8 agonist, rather than to TLR-4 activation caused by lipopolysaccharide (LPS) (Table S2, ESI†).^40^

### AAA-conjugates induce *ex vivo* antigen-specific T cell activation

**Fig. 3:**
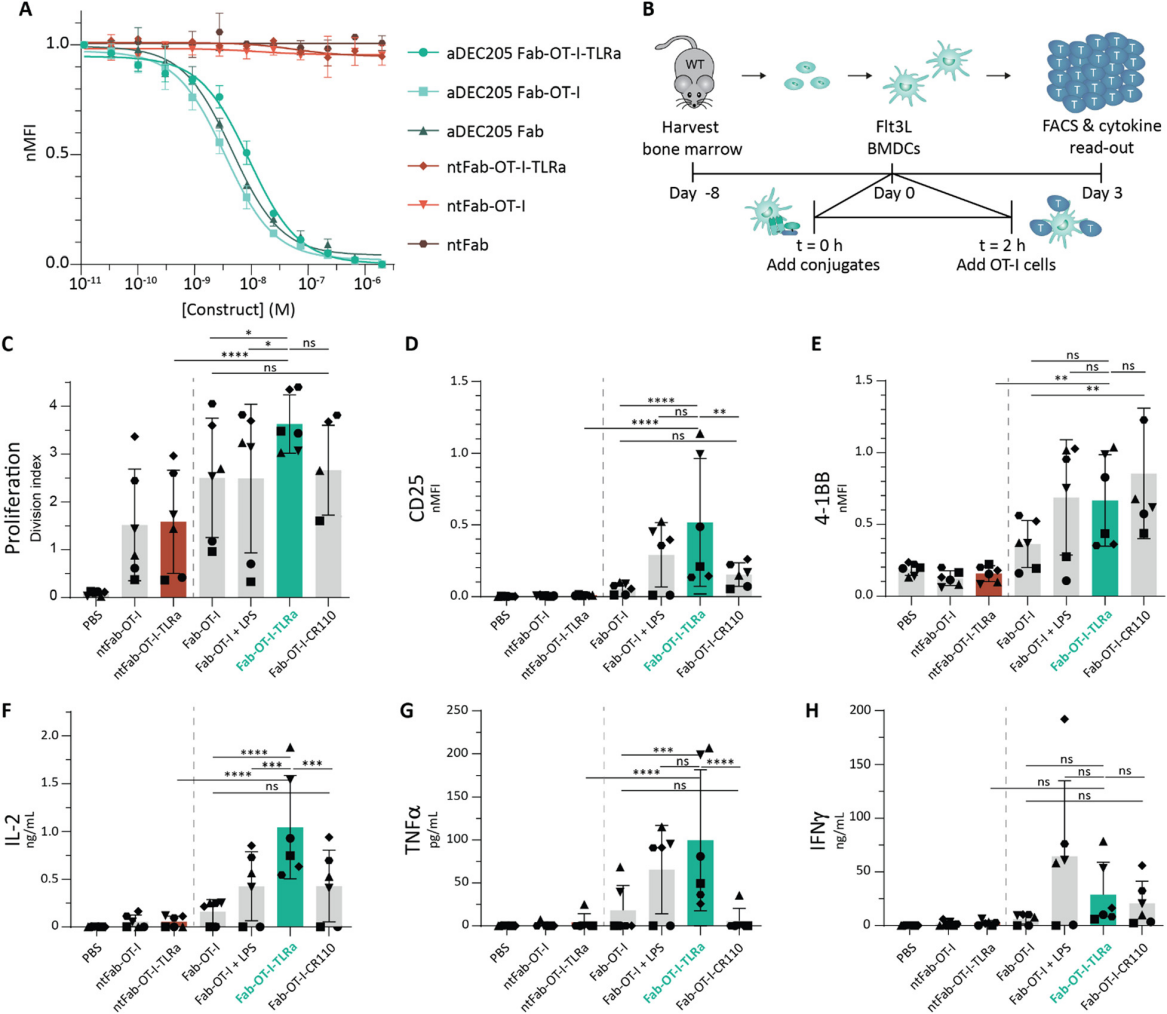
*Ex vivo* analysis of conjugates. (A) Competitive binding assay of conjugates against parental mAb (NLDC-145). Data (*n* = 3 for aDEC205 conjugates, *n* = 2 for non-targeting conjugates) are shown as mean fluorescence intensity ± SD normalized to highest signal intensity with a one site - fit log IC_50_ least squares fit curve. (B) Schematic overview of *ex vivo* T cell activation assay. In short, Flt3L-BMDCs were generated and pulsed for 2 h with 10 nM conjugate. Sequentially, the BMDCs were washed and a co-culture of BMDCs and OT-I cells (1 : 5 ratio) was set-up. After 3 days, OT-I cells were analyzed using FACS and cytokines in the supernatant were assessed *via* ELISA. (C)–(E) Flow cytometry analysis of OT-I cells. Data (*n* = 6, technical duplicates) are depicted as division index (C) and as mean fluorescence intensity normalized to positive control ± SD for CD25 (D) and 4-1BB (E). Statistical significance using one-way ANOVA with Sidak's multiple comparison correction is depicted as ****P < 0.0001, ***P < 0.001, **P < 0.01, *P < 0.05, ns *P* > 0.05. (F)–(H) ELISA analysis (*n* = 6, technical duplicates) of IL-2 (F), TNFα (G), and IFNγ (H). Data is depicted as mean ± SD. Statistical significance using one-way ANOVA with Sidak's multiple comparison correction is depicted as ****P < 0.0001, ***P < 0.001, **P < 0.01, *P < 0.05, ns *P* > 0.05.

Next, we tested the binding capacity and functionality of our AAA-conjugates. DEC205 binding was assessed in a flow cytometry-based competitive binding assay using DEC205-expressing JAWS II cells (a mouse immature DC line) (Fig. 3A). In short, JAWS II cells were incubated with our conjugates, followed by incubation with a commercially available fluorescently labeled aDEC205 mAb of the same clone (NLDC-145). Comparable competitive binding curves were obtained for the unaltered Fab fragment, antigen-labeled Fab fragment (Fab-OT-I) and AAA-conjugate (Fab-OT-I-TLRa). Next to that, no binding was observed for non-targeting controls. This indicates no decrease in DEC205 affinity for the conjugates in which the cargo is conjugated site-specifically, away from the binding domain of the Fab fragment.

To assess the function of the conjugates, a DC-mediated antigen-specific T cell activation assay was set-up using OT-I cells (Fig. 3B). OT-I cells are CD8^+^ T cells which specifically recognize presentation of the SIINFEKL-epitope in H-2Kb MHC I complexes.^36^ We generated Flt3L bone-marrow derived DCs (BMDCs) from female wild type C57BL/6J mice, which were subsequently pulsed with conjugates (10 nM) for 2 h, washed and co-cultured with CellTrace™-labeled OT-I cells.^41^ After 3 days we assessed T cell activation by measuring proliferation (division index), expression of activation markers (CD25, 4-1BB), and secretion of proinflammatory cytokines (IL-2, TNFα, IFNγ) (Fig. 3C–H). The first thing that became evident from the data is the crucial importance of targeting the antigen to DEC205, since in all conditions the non-targeted controls induced significantly less T cell activation. Co-targeting of the TLR-7/8 agonist by the AAA-conjugate (Fab-OT-I-TLRa) induced the highest OT-I T cell activation, comparable to Fab-OT-I adjuvated with LPS, a potent TLR-4 agonist used as positive control. The beneficial effect of co-targeting TLRa was most notable in the expression of CD25, as well as IL-2 and TNFα secretion. IFNγ secretion was higher with Fab-OT-I adjuvated with LPS, as expected by triggering TLR-4.^42^

Because the TLRa is hydrophobic, we attached fluorophores to Fab-OT-I to control for potential differences in construct uptake, routing and/or processing. Interestingly, fluorescent labels attached to Fab-OT-I (Fab-OT-I-CR110 and Fab-OT-I-AF488 (Fig. S4, ESI†)) enhanced 4-1BB expression, yet no such increase was observed for CD25 or TNFα. 4-1BB expression is associated with T cell receptor (TCR) signaling, also independent of co-stimulation. This demonstrates that fluorescent labeling of antigenic molecules significantly alters antigen presentation by BMDCs.^43^ We hypothesize that the hydrophobic character of fluorophores, as well as TLRa, enhances endosomal escape of the antigenic conjugates.^44,45^ In turn, this leads to higher levels of cross-presentation of the epitopes. Yet, AAA-conjugate (Fab-OT-I-TLRa) treatment induces higher expression of CD25, IL-2 and TNFα, which is indicative of TLR-7/8 mediated DC maturation. The beneficial effect of co-delivery of the TLR agonist becomes apparent when reaching limiting amounts of antigen, as higher, saturating antigen concentrations mask the effect (Fig. S5, ESI†). In summary, the results confirm that targeting DEC205 improves antigen presentation and provides a proof-of-concept for enhanced T cell activation through co-delivery of antigen and adjuvant by the AAA-conjugates.

### Subcutaneous injection of AAA-conjugates does not enhance OT-I activation

**Fig. 4:**
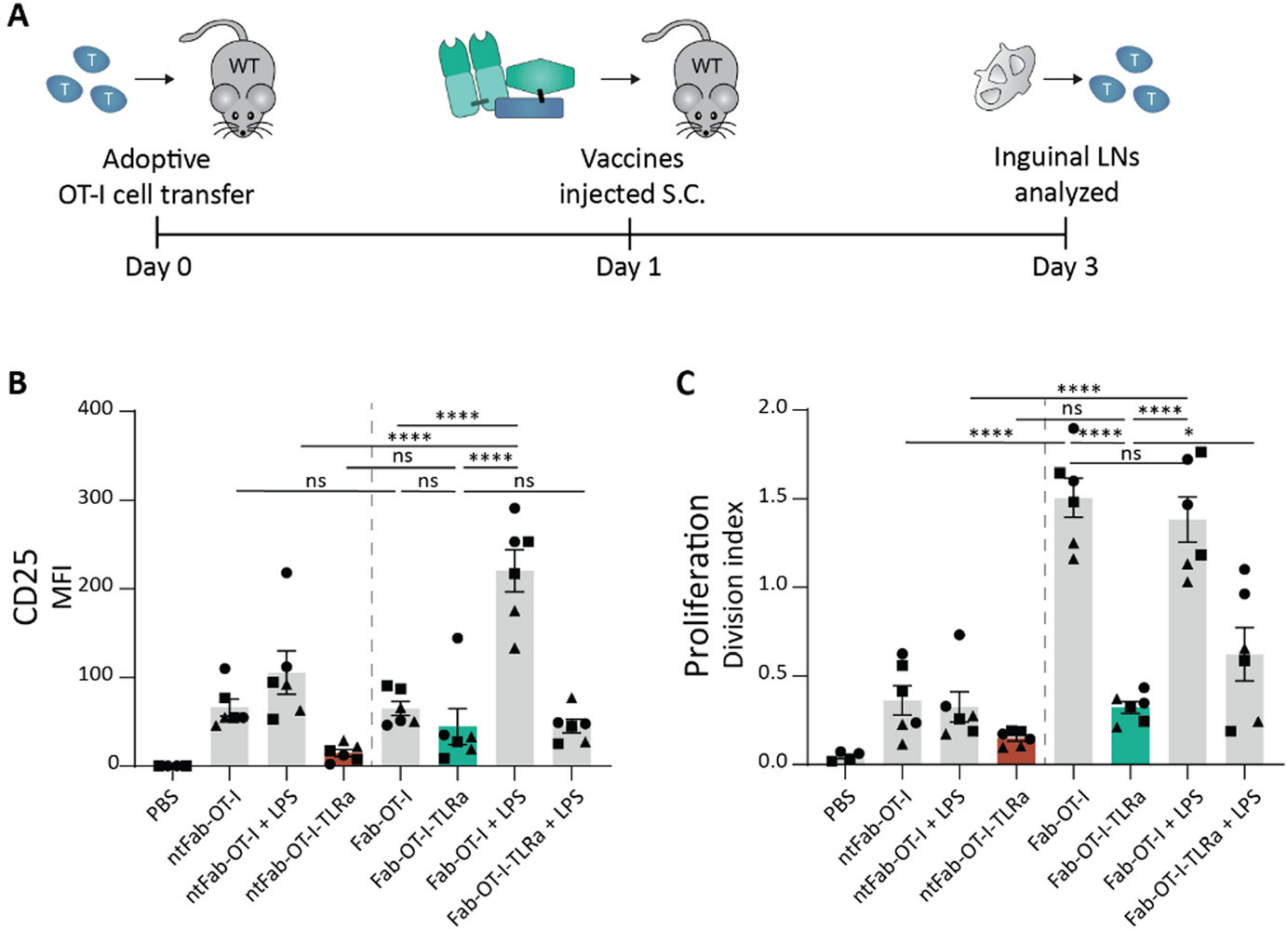
*In vivo* OT-I cell activation assay after subcutaneous injection of conjugates. (A) Schematic representation of *in vivo* assay. In short, 8–12 week old female wildtype C57BL/6j mice received 1 × 10^6^ CTV-labeled OT-I cells and the following day were injected (s.c.) with the vaccine conjugates supplemented with 10 μg LPS if indicated. 2 days later, inguinal lymph nodes and spleen were harvested and analyzed using flow cytometry. (B) and (C) Flow cytometry analysis of OT-I cells. Data (*n* = 3, left and right inguinal lymph node shown individually) are depicted as mean fluorescence intensity ± SEM for CD25 (B) and division index ± SEM for proliferation (C). Statistical significance using one-way ANOVA with Sidak's multiple comparison correction is depicted as ****P < 0.0001, ***P < 0.001, **P < 0.01, *P < 0.05, ns *P* > 0.05.

Encouraged by the results obtained *ex vivo*, we explored the efficacy of AAA-conjugates *in vivo*. We adoptively transferred wild type C57BL/6 mice with CellTrace™-labeled OT-I cells and injected our conjugates subcutaneously the following day (Fig. 4A). We opted for subcutaneous (s.c.) injection, because of the high DEC205 expression of skin DCs that can capture antigens of s.c. injected vaccines.^22,46,47^ Two days after vaccination, the inguinal lymph nodes were harvested, and OT-I cell proliferation and CD25 expression were assessed by flow cytometry. The highest OT-I proliferation was observed in both DEC205-targeted Fab-OT-I treatment conditions, irrespective of LPS co-administration (Fig. 4B and C), however CD25 expression, indicative of true T cell activation, required LPS. Unlike *ex vivo*, Fab-OT-I-TLRa did not induce significant OT-I cell proliferation or activation, similar to non-targeting controls. We confirmed that the construct remained stable after incubation in mouse serum (Fig. S6, ESI†). These findings combined strongly indicate that Fab-OT-I-TLRa does not reach DCs to a similar degree as Fab-OT-I when injected subcutaneously. LPS co-injection does not increase T cell activation suggesting that the small fraction that does reach DCs is able to activate these DCs. We hypothesized the hydrophobic character of the AAA-conjugate could explain the suboptimal *in vivo* performance. Thus, we analyzed the change in hydrophobicity as a consequence of each consecutive protein modification by reversed phase-high-performance liquid chromatography (RP-HPLC). We observed an increase in retention time upon attachment of the antigen compared to the starting Fab fragment (Fig. S8, ESI†), which was further increased upon the sequential attachment of the DBCO-TLRa to generate the AAA-conjugate. This upward shift in retention time demonstrates increased hydrophobicity, as a consequence of the TLRa in combination with the epitope. We speculate that the increased hydrophobicity results in suboptimal pharmacokinetic and pharmacodynamic (PK/PD) properties detrimental for vaccine efficacy.

### Separated targeted delivery of antigen and adjuvant is capable of inducing strong T cell proliferation *in vivo*

**Fig. 5:**
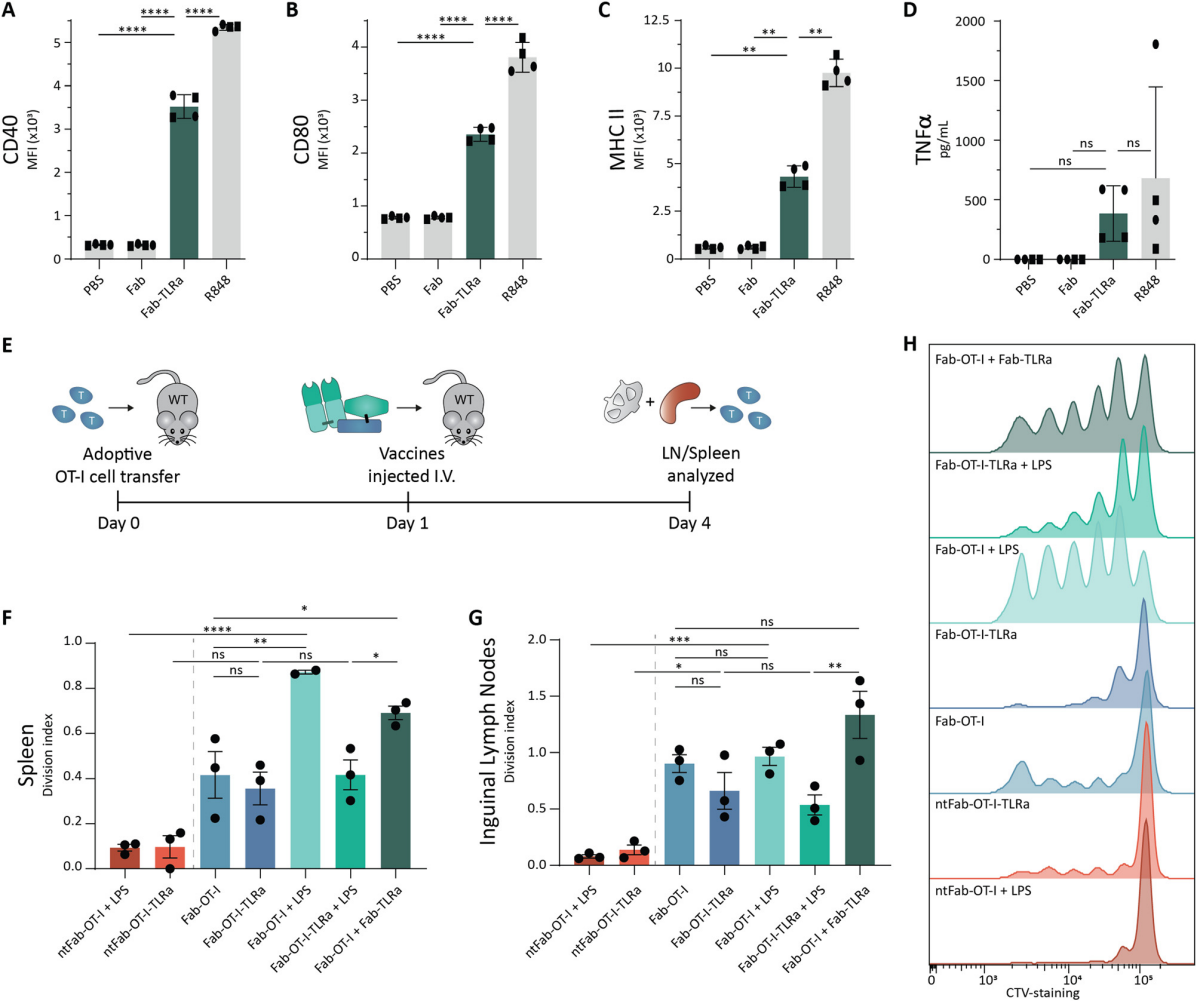
*Ex vivo* and *in vivo* activation assays for targeted adjuvant. (A)–(C) Flow cytometry analysis of Flt3L BMDCs pulsed overnight with 1 μM adjuvant. Data (*n* = 4, 2 donors) are depicted as mean fluorescence intensity ± SD for CD40 (A), CD80 (B), and MHC II (C). Statistical significance using one-way ANOVA with Tukey's multiple comparison correction is depicted as ****P < 0.0001, ***P < 0.001, **P < 0.01, *P < 0.05, ns *P* > 0.05. (D) TNFα ELISA analysis of Flt3L BMDCs pulsed overnight at 1 μM adjuvant. Data (*n* = 4, 2 donors) are depicted as mean ± SD. Statistical significance using one-way ANOVA with Tukey's multiple comparison correction is depicted as ****P < 0.0001, ***P < 0.001, **P < 0.01, *P < 0.05, ns *P* > 0.05. (E) Schematic representation of *in vivo* assay. In short, 8–12 week old female wildtype C57BL/6j mice received 1 × 10^6^ CTV-labeled OT-I cells and the following day were injected (i.v.) with the vaccine conjugates. 3 days later, inguinal lymph nodes and spleen were harvested and analyzed using flow cytometry. (F)–(H) Flow cytometry analysis of OT-I cells. Data (*n* = 3) are depicted as division index ± SEM for OT-I cells isolated from spleen (F) and inguinal lymph node (G). Representative histograms for proliferation of OT-I cells in the spleen are shown (H). Statistical significance using one-way ANOVA with Sidak's multiple comparison correction is depicted as ****P < 0.0001, ***P < 0.001, **P < 0.01, *P < 0.05, ns *P* > 0.05.

As we observed potent T cell activation when Fab-OT-I was adjuvated with LPS, we reasoned that separate targeted delivery of antigen and adjuvant could result in a less hydrophobic conjugate, while maintaining the benefits of targeted adjuvant delivery (Fig. 1A). Therefore, we attached the same DBCO-modified TLRa as used before to the aDEC205 Fab fragment after site-specifically attaching GGGK(N_3_) to generate Fab-TLRa (Fig. S7, ESI†). RP-HPLC analysis (Fig. S8, ESI†) indicated the a lower retention time for the Fab-TLRa compared to the AAA-conjugate, suggesting reduced hydrophobicity. We assessed the functionality of the TLRa after conjugation by incubating Flt3L BMDCs overnight with 1 μM Fab-TLRa (Fig. 5A–D). Although less potent then free R848, Fab-TLRa was capable of inducing the upregulation of the DC maturation markers CD40, CD80, and MHC II, as well as inducing TNFα secretion. This indicates that the conjugated adjuvant can mature and activate antigen-presenting cells, which prompted us to assess the potential of separate targeted delivery of antigen and adjuvant for antigen-specific T cell activation *in vivo*.

For these experiments, we adjusted the route of administration from s.c. to intravenous (i.v.) injection. We reasoned that injection into blood circulation avoids s.c. depot formation and could result in better PK/PD profiles leading to improved systemic distribution.^48,49^ Wild type mice received CellTrace™-labeled OT-I cells, followed by the vaccines the next day. After 3 days, spleen and inguinal lymph nodes were harvested (Fig. 5E). Targeting of the antigen appeared crucial for T cell activation, as non-targeting control conjugates induced minimal proliferation (Fig. 5F–H), in line with the *ex vivo* experiments (Fig. 3). The AAA-conjugate (Fab-OT-I-TLRa) demonstrated moderate T cell activation, to a similar degree as the non-adjuvated Fab-OT-I (Fig. 5F–H), indicating that upon i.v. administration the AAA-conjugates reach DCs. Co-administration of LPS enhanced T cell proliferation in the spleen induced by Fab-OT-I, whereas this had no effect in the case of Fab-OT-I-TLRa. This result could indicate the self-supporting capability of the AAA-conjugate for DC activation. Importantly, co-administration of DEC205-targeted adjuvant (Fab-TLRa) and Fab-OT-I resulted in significantly enhanced OT-I T cell activation compared to non-adjuvanted Fab-OT-I and Fab-OT-I-TLRa. This supports the hypothesis that, in the format investigated in this work, separate targeted delivery of adjuvant and antigen has better efficacy than the triple conjugate and demonstrates targeting adjuvant to DCs is a feasible approach for the improvement of cancer vaccines.

## Discussion

In this study, we provide the first example of the engineering of a molecularly defined antibody-fragment, antigen and small molecule adjuvant conjugate for DC-targeted co-delivery. *Ex vivo* T cell activation assays showed a significantly enhanced DC-mediated T cell proliferation induced by the AAA-conjugate compared to the non-adjuvated antibody fragment–antigen conjugate. These results support the further development of targeted antigen–adjuvant conjugates in the context of cancer vaccines. Interestingly, not only the AAA-conjugate, but also the fluorescently labeled control conjugates improved T cell proliferation *ex vivo*. We hypothesize that the hydrophobic moiety attached close to the antigen improves cross-presentation of the antigen. This would be a consequence of the hydrophobic moieties improving membrane permeability to facilitate endosomal escape.^44,45^ This finding argues for caution of attaching fluorophores when studying antigen processing, as the fluorophore significantly alters intracellular localization. This may also be explored to promote antigen presentation in the context of DC-targeted antigen delivery.

Unfortunately, the *ex vivo* performance did not translate to *in vivo* efficacy of the AAA-conjugate. We hypothesize the hydrophobic character of the AAA-conjugate caused by the adjuvant as well as the CD8-epitope, results in an unfavorable pharmacokinetic profile. To attain effective targeted co-delivery *in vivo*, efforts should be made to improve water-solubility and thereby bioavailability of the conjugates. One strategy may be to PEGylate the conjugate to improve solubility. Recently, we demonstrated that this is effective to attach the insoluble cancer testis antigen epitope NY-ESO-1 (157-165) to the chemokine XCL1 for targeted delivery to human conventional type 1 dendritic cells (cDC1s).^50^ PEGylation can induce anti-PEG antibodies *in vivo*, preventing repeated administration of the vaccine. To avoid this, branched PEG-chains are recommended as they significantly lower the induction of anti-PEG antibodies *in vivo.*^51^ Alternatively, the biophysical properties of the conjugate can be enhanced by exchanging the relatively large, hydrophobic DBCO-group for a smaller, more hydrophilic linker. Bicyclo[6.1.0]non-4-yne (BCN) is a strained alkyne that is more hydrophilic in exchange for lower reactivity compared to DBCO and provides an attractive substitute.^52^ A different approach is exchange of the azido-lysine into an oxo-lysine. This enables oxime ligation, which results in a smaller footprint of the linker compared to SPAAC.^53^ This strategy has been used in combination with sortase-mediated conjugation to prepare DNA–nanobody conjugates and may be applied for the generation of AAA-conjugates as well.^54^

In this study, we used an imiquimod-derivative to stimulate TLR-7/8, because of their synthetic accessibility and clinical use as antiviral agents.^38,39^ Furthermore, TLR-7 and TLR-8 both localize in endosomal compartments, similar to TLR-3 and TLR-9.^55^ This makes agonists of these TLRs attractive targets for co-delivery with antigens, as it ensures DC maturation only after simultaneous uptake of the antigen. Poly(I:C), a mismatched double stranded RNA, is a potent TLR-3 agonist and clinically explored as it has been shown to enhance cytotoxic T cell responses.^56,57^ Examples of site-specific conjugation of poly(I:C) are scarce, but could potentially be achieved through phosphoramidite chemistry on either terminus of the RNA chains.^18^ CU-CPT17e is a small molecule multi-TLR agonist that is reported to activate TLR-3/7/8, although its potency is not on par with poly(I:C).^58^ It might be a starting point for the development of more potent conjugatable small molecule TLR-3 agonists. TLR-2/6 agonists have been used successfully in Phase I clinical trials attached to HPV-16 E6 synthetic long peptides.^11^ These hydrophobic moieties may be challenging to ligate, yet have proven to be safe and effective in patients. Ultimately, multiple different TLR agonists could be incorporated into a single DC-targeted molecule, either together with a tumor antigen, or separately targeted, which is expected to result in potent DC activating conjugates.^59,60^

Finally, to ensure clinical translation, special consideration is required for the ease of preparation. Tumors are heterogenic and a single epitope will not suffice to induce complete tumor regression.^61^ Therefore, the flexibility to easily conjugate different antigens is critical to deliver a successful targeted cancer vaccine. An off-the-shelf product can be created using a library of established tumor-associated antigens.^62^ Alternatively, a personalized medicine approach can be used through screening for tumor-specific neoepitopes.^63,64^ The latter approach is likely to be more effective, yet will also be more laborious. Of the two DC targeting approaches – the AAA- and the “2× AA-conjugate” approach – presented in this work, the co-administration of separate DC-targeted adjuvant and antigen conjugates would be most feasible for future therapeutic applications, because an off-the-shelf targeted adjuvant can be combined with a personalized DC-targeted antigen conjugate. The observed capacity of induction of T cell proliferation (Fig. 5) of the “2× AA-conjugate” approach argues for DC-targeted delivery of adjuvant in future applications opposed to the currently used systemic adjuvant administration. Next to CD8-epitopes, CD4-epitopes are essential to induce full blown antitumor immune responses.^65,66^ Although CD4-neoepitopes are more difficult to predict due to the relative shallow binding cleft of MHC II complexes, new approaches for prediction and discovery are in development.^67–69^ Dosing and specific targeting is particularly important for CD4-epitope delivery, as too high doses could suppress tumor immunity *via* antigen-specific killing of cDC1s by cytotoxic Tr1 CD4^+^ T cells.^7^ By separating CD8- and CD4-epitopes on different targeting moieties, the ratio of CD8–CD4 antigen delivery can be controlled.

## Conclusions

To conclude, we present a single molecule DC-targeted antigen–adjuvant conjugate, coined AAA-conjugate, generated site-specifically utilizing a combination of sortase-mediated chemoenzymatic ligation and SPAAC. Targeted co-delivery of antigen and adjuvant with our AAA-conjugate enhanced T cell activation *ex vivo* due to a combination of DC maturation and enhanced cross-presentation by the hydrophobic character of the TLR-agonist. The *ex vivo* efficacy of the AAA-conjugate did not translate to *in vivo*, but the data suggests that AAA-conjugates are self-adjuvating, since co-administering LPS did not enhance T cell proliferation. *In vivo* T cell activation could be increased by separating adjuvant and antigen onto two different DEC205-targeting conjugates to overcome the unfavorable hydrophobic character of the AAA-conjugate. In conclusion, while suboptimal biophysical properties are a remaining issue for single molecule AAA-conjugates, we provide a proof-of-concept for a modular platform for DC-targeted co-delivery of antigen and adjuvant, expanding the chemical toolbox for the development of therapeutic vaccines.

## Author contributions

ZW conceived and designed the study, performed and analyzed experiments, visualized and wrote the paper. IRT designed and prepared conjugates. CL designed, optimized, and performed animal experiments. EvD and DvD performed protein production and isolation. RP assisted in RP-HPLC analysis. FT, SM, IH, and FvD synthesized molecules used in this study. RC and KS assisted in the animal experiments. Fvd, FF and JvS assisted in experimental design and optimization. CF, AE, and FS assisted in supervision of the study. MV conceived and supervised the study, wrote the paper and acquired funding.

## Data availability

The main data supporting the results of this study are available within the paper and its ESI.† Raw data for this study is available at the Radboud Data Repository (RDR) at https://doi.org/10.34973/cfvx-0443.

## Conflicts of interest

There are no conflicts to declare.

## Supplementary Material

CB-OLF-D5CB00014A-s001

## References

[cit1] Sellars M. C., Wu C. J., Fritsch E. F. (2022). Cancer vaccines: Building a bridge over troubled waters. Cell.

[cit2] Saxena M., van der Burg S. H., Melief C. J. M., Bhardwaj N. (2021). Therapeutic cancer vaccines. Nat. Rev. Cancer.

[cit3] Toes R. E., Offringa R., Blom R. J., Melief C. J., Kast W. M. (1996). Peptide vaccination can lead to enhanced tumor growth through specific T-cell tolerance induction. Proc. Natl. Acad. Sci. U. S. A..

[cit4] Welters M. J. P., Kenter G. G., Piersma S. J., Vloon A. P. G., Löwik M. J. G., Berends-van der Meer D. M. A., Drijfhout J. W., Valentijn A. R. P. M., Wafelman A. R., Oostendorp J., Fleuren G. J., Offringa R., Melief C. J. M., van der Burg S. H. (2008). Induction of Tumor-Specific CD4+ and CD8+ T-Cell Immunity in Cervical Cancer Patients by a Human Papillomavirus Type 16 E6 and E7 Long Peptides Vaccine. Clin. Cancer Res..

[cit5] Billeskov R., Wang Y., Solaymani-Mohammadi S., Frey B., Kulkarni S., Andersen P., Agger E. M., Sui Y., Berzofsky J. A. (2017). Low Antigen Dose in Adjuvant-Based Vaccination Selectively Induces CD4 T Cells with Enhanced Functional Avidity and Protective Efficacy. J. Immunol..

[cit6] Alexander-Miller M. A., Leggatt G. R., Sarin A., Berzofsky J. A. (1996). Role of antigen, CD8, and cytotoxic T lymphocyte (CTL) avidity in high dose antigen induction of apoptosis of effector CTL. J. Exp. Med..

[cit7] Sultan H., Takeuchi Y., Ward J. P., Sharma N., Liu T.-T., Sukhov V., Firulyova M., Song Y., Ameh S., Brioschi S., Khantakova D., Arthur C. D., White J. M., Kohlmiller H., Salazar A. M., Burns R., Costa H. A., Moynihan K. D., Yeung Y. A., Djuretic I., Schumacher T. N., Sheehan K. C. F., Colonna M., Allison J. P., Murphy K. M., Artyomov M. N., Schreiber R. D. (2024). Neoantigen-specific cytotoxic Tr1 CD4 T cells suppress cancer immunotherapy. Nature.

[cit8] Toes R. E., van der Voort E. I., Schoenberger S. P., Drijfhout J. W., van Bloois L., Storm G., Kast W. M., Offringa R., Melief C. J. (1998). Enhancement of tumor outgrowth through CTL tolerization after peptide vaccination is avoided by peptide presentation on dendritic cells. J. Immunol..

[cit9] Wilson N. S., Behrens G. M. N., Lundie R. J., Smith C. M., Waithman J., Young L., Forehan S. P., Mount A., Steptoe R. J., Shortman K. D., de Koning-Ward T. F., Belz G. T., Carbone F. R., Crabb B. S., Heath W. R., Villadangos J. A. (2006). Systemic activation of dendritic cells by Toll-like receptor ligands or malaria infection impairs cross-presentation and antiviral immunity. Nat. Immunol..

[cit10] Heikenwalder M., Polymenidou M., Junt T., Sigurdson C., Wagner H., Akira S., Zinkernagel R., Aguzzi A. (2004). Lymphoid follicle destruction and immunosuppression after repeated CpG oligodeoxynucleotide administration. Nat. Med..

[cit11] Speetjens F. M., Welters M. J. P., Slingerland M., van Poelgeest M. I. E., de P. J., van Steenwijk V., Roozen I., Boekestijn S., Loof N. M., Zom G. G., Valentijn A. R. P. M., Krebber W.-J., Meeuwenoord N. J., Janssen C. A. H., Melief C. J. M., van der Marel G. A., Filippov D. V., van der Burg S. H., Gelderblom H., Ossendorp F. (2022). Intradermal vaccination of HPV-16 E6 synthetic peptides conjugated to an optimized Toll-like receptor 2 ligand shows safety and potent T cell immunogenicity in patients with HPV-16 positive (pre-)malignant lesions. J. Immunother. Cancer.

[cit12] Lynn G. M., Sedlik C., Baharom F., Zhu Y., Ramirez-Valdez R. A., Coble V. L., Tobin K., Nichols S. R., Itzkowitz Y., Zaidi N., Gammon J. M., Blobel N. J., Denizeau J., de la Rochere P., Francica B. J., Decker B., Maciejewski M., Cheung J., Yamane H., Smelkinson M. G., Francica J. R., Laga R., Bernstock J. D., Seymour L. W., Drake C. G., Jewell C. M., Lantz O., Piaggio E., Ishizuka A. S., Seder R. A. (2020). Peptide-TLR-7/8a conjugate vaccines chemically programmed for nanoparticle self-assembly enhance CD8 T-cell immunity to tumor antigens. Nat. Biotechnol..

[cit13] Ilyinskii P. O., Roy C. J., O’Neil C. P., Browning E. A., Pittet L. A., Altreuter D. H., Alexis F., Tonti E., Shi J., Basto P. A., Iannacone M., Radovic-Moreno A. F., Langer R. S., Farokhzad O. C., von Andrian U. H., Johnston L. P. M., Kishimoto T. K. (2014). Adjuvant-carrying synthetic vaccine particles augment the immune response to encapsulated antigen and exhibit strong local immune activation without inducing systemic cytokine release. Vaccine.

[cit14] Blander J. M., Medzhitov R. (2004). Regulation of phagosome maturation by signals from toll-like receptors. Science.

[cit15] Hoffmann E., Kotsias F., Visentin G., Bruhns P., Savina A., Amigorena S. (2012). Autonomous phagosomal degradation and antigen presentation in dendritic cells. Proc. Natl. Acad. Sci. U. S. A..

[cit16] Andrews C. D., Huh M.-S., Patton K., Higgins D., Van Nest G., Ott G., Lee K.-D. (2012). Encapsulating Immunostimulatory CpG Oligonucleotides in Listeriolysin O-Liposomes Promotes a Th1-Type Response and CTL Activity. Mol. Pharmaceutics.

[cit17] Mitchell M. J., Billingsley M. M., Haley R. M., Wechsler M. E., Peppas N. A., Langer R. (2021). Engineering precision nanoparticles for drug delivery. Nat. Rev. Drug Discovery.

[cit18] Ignacio B. J., Albin T. J., Esser-Kahn A. P., Verdoes M. (2018). Toll-like Receptor Agonist Conjugation: A Chemical Perspective. Bioconjugate Chem..

[cit19] Jahanbani S., Hansen P. S., Blum L. K., Bastounis E. E., Ramadoss N. S., Pandrala M., Kirschmann J. M., Blacker G. S., Love Z. Z., Weissman I. L., Nemati F., Tal M. C., Robinson W. H. (2023). Increased macrophage phagocytic activity with TLR9 agonist conjugation of an anti- *Borrelia burgdorferi* monoclonal antibody. Clin. Immunol..

[cit20] Gadd A. J. R., Greco F., Cobb A. J. A., Edwards A. D. (2015). Targeted Activation of Toll-Like Receptors: Conjugation of a Toll-Like Receptor 7 Agonist to a Monoclonal Antibody Maintains Antigen Binding and Specificity. Bioconjugate Chem..

[cit21] Fang S., Brems B. M., Olawode E. O., Miller J. T., Brooks T. A., Tumey L. N. (2022). Design and Characterization of Immune-Stimulating Imidazo[4,5-c]quinoline Antibody-Drug Conjugates. Mol. Pharmaceutics.

[cit22] Bonifaz L., Bonnyay D., Mahnke K., Rivera M., Nussenzweig M. C., Steinman R. M. (2002). Efficient targeting of protein antigen to the dendritic cell receptor DEC-205 in the steady state leads to antigen presentation on major histocompatibility complex class I products and peripheral CD8+ T cell tolerance. J. Exp. Med..

[cit23] Bonifaz L. C., Bonnyay D. P., Charalambous A., Darguste D. I., Fujii S.-I., Soares H., Brimnes M. K., Moltedo B., Moran T. M., Steinman R. M. (2004). In vivo targeting of antigens to maturing dendritic cells via the DEC-205 receptor improves T cell vaccination. J. Exp. Med..

[cit24] Wijfjes Z., van Dalen F. J., Le Gall C. M., Verdoes M. (2023). Controlling Antigen Fate in Therapeutic Cancer Vaccines by Targeting Dendritic Cell Receptors. Mol. Pharmaceutics.

[cit25] Kreutz M., Giquel B., Hu Q., Abuknesha R., Uematsu S., Akira S., Nestle F. O., Diebold S. S. (2012). Antibody-Antigen-Adjuvant Conjugates Enable Co-Delivery of Antigen and Adjuvant to Dendritic Cells in Cis but Only Have Partial Targeting Specificity. PLoS One.

[cit26] Arabpour M., Paul S., Grauers Wiktorin H., Kaya M., Kiffin R., Lycke N., Hellstrand K., Martner A. (2022). An adjuvant-containing cDC1-targeted recombinant fusion vaccine conveys strong protection against murine melanoma growth and metastasis. OncoImmunology.

[cit27] Schmitt S., Tahk S., Lohner A., Hänel G., Maiser A., Hauke M., Patel L., Rothe M., Josenhans C., Leonhardt H., Griffioen M., Deiser K., Fenn N. C., Hopfner K.-P., Subklewe M. (2020). Fusion of Bacterial Flagellin to a Dendritic Cell-Targeting αCD40 Antibody Construct Coupled With Viral or Leukemia-Specific Antigens Enhances Dendritic Cell Maturation and Activates Peptide-Responsive T Cells. Front. Immunol..

[cit28] Jeon D., Hill E., McNeel D. G. (2024). Toll-like receptor agonists as cancer vaccine adjuvants. Hum. Vaccines Immunother..

[cit29] Shen B.-Q., Xu K., Liu L., Raab H., Bhakta S., Kenrick M., Parsons-Reponte K. L., Tien J., Yu S.-F., Mai E., Li D., Tibbitts J., Baudys J., Saad O. M., Scales S. J., McDonald P. J., Hass P. E., Eigenbrot C., Nguyen T., Solis W. A., Fuji R. N., Flagella K. M., Patel D., Spencer S. D., Khawli L. A., Ebens A., Wong W. L., Vandlen R., Kaur S., Sliwkowski M. X., Scheller R. H., Polakis P., Junutula J. R. (2012). Conjugation site modulates the in vivo stability and therapeutic activity of antibody-drug conjugates. Nat. Biotechnol..

[cit30] Strop P., Liu S.-H., Dorywalska M., Delaria K., Dushin R. G., Tran T.-T., Ho W.-H., Farias S., Casas M. G., Abdiche Y., Zhou D., Chandrasekaran R., Samain C., Loo C., Rossi A., Rickert M., Krimm S., Wong T., Chin S. M., Yu J., Dilley J., Chaparro-Riggers J., Filzen G. F., O’Donnell C. J., Wang F., Myers J. S., Pons J., Shelton D. L., Rajpal A. (2013). Location Matters: Site of Conjugation Modulates Stability and Pharmacokinetics of Antibody Drug Conjugates. Chem. Biol..

[cit31] van der Schoot J. M. S., Fennemann F. L., Valente M., Dolen Y., Hagemans I. M., Becker A. M. D., Le Gall C. M., van Dalen D., Cevirgel A., van Bruggen J. A. C., Engelfriet M., Caval T., Bentlage A. E. H., Fransen M. F., Nederend M., Leusen J. H. W., Heck A. J. R., Vidarsson G., Figdor C. G., Verdoes M., Scheeren F. A. (2019). Functional diversification of hybridoma-produced antibodies by CRISPR/HDR genomic engineering. Sci. Adv..

[cit32] Strohalm M., Kavan D., Novák P., Volný M., Havlíček V. (2010). mMass 3: A Cross-Platform Software Environment for Precise Analysis of Mass Spectrometric Data. Anal. Chem..

[cit33] Wang B., Zaidi N., He L.-Z., Zhang L., Kuroiwa J. M., Keler T., Steinman R. M. (2012). Targeting of the non-mutated tumor antigen HER2/neu to mature dendritic cells induces an integrated immune response that protects against breast cancer in mice. Breast Cancer Res..

[cit34] Liu H., May K. (2012). Disulfide bond structures of IgG molecules. MAbs.

[cit35] Dorr B. M., Ham H. O., An C., Chaikof E. L., Liu D. R. (2014). Reprogramming the specificity of sortase enzymes. Proc. Natl. Acad. Sci. U. S. A..

[cit36] Hogquist K. A., Jameson S. C., Heath W. R., Howard J. L., Bevan M. J., Carbone F. R. (1994). T cell receptor antagonist peptides induce positive selection. Cell.

[cit37] Swee L. K., Guimaraes C. P., Sehrawat S., Spooner E., Barrasa M. I., Ploegh H. L. (2013). Sortase-mediated modification of αDEC205 affords optimization of antigen presentation and immunization against a set of viral epitopes. Proc. Natl. Acad. Sci. U. S. A..

[cit38] Shukla N. M., Mutz C. A., Ukani R., Warshakoon H. J., Moore D. S., David S. A. (2010). Syntheses of fluorescent imidazoquinoline conjugates as probes of Toll-like receptor 7. Bioorg. Med. Chem. Lett..

[cit39] Bhagchandani S., Johnson J. A., Irvine D. J. (2021). Evolution of Toll-like receptor 7/8 agonist therapeutics and their delivery approaches: From antiviral formulations to vaccine adjuvants. Adv. Drug Delivery Rev..

[cit40] Alexander C., Rietschel E. T. (2001). Bacterial lipopolysaccharides and innate immunity. J. Endotoxin Res..

[cit41] Naik S. H., Proietto A. I., Wilson N. S., Dakic A., Schnorrer P., Fuchsberger M., Lahoud M. H., O’Keeffe M., Shao Q., Chen W., Villadangos J. A., Shortman K., Wu L. (2005). Cutting Edge: Generation of Splenic CD8+ and CD8− Dendritic Cell Equivalents in Fms-Like Tyrosine Kinase 3 Ligand Bone Marrow Cultures. J. Immunol..

[cit42] Lu Y.-C., Yeh W.-C., Ohashi P. S. (2008). LPS/TLR4 signal transduction pathway. Cytokine.

[cit43] Choi B. K., Lee H.-W. (2020). The Murine CD137/CD137 Ligand Signalosome: A Signal Platform Generating Signal Complexity. Front. Immunol..

[cit44] Naylor M. R., Ly A. M., Handford M. J., Ramos D. P., Pye C. R., Furukawa A., Klein V. G., Noland R. P., Edmondson Q., Turmon A. C., Hewitt W. M., Schwochert J., Townsend C. E., Kelly C. N., Blanco M.-J., Lokey R. S. (2018). Lipophilic Permeability Efficiency Reconciles the Opposing Roles of Lipophilicity in Membrane Permeability and Aqueous Solubility. J. Med. Chem..

[cit45] Liu X., Testa B., Fahr A. (2011). Lipophilicity and Its Relationship with Passive Drug Permeation. Pharm. Res..

[cit46] Inaba K., Swiggard W. J., Inaba M., Meltzer J., Miryza A., Sasagawa T., Nussenzweig M. C., Steinman R. U. (1995). Tissue Distribution of the DEC-205 Protein That Is Detected by the Monoclonal Antibody NLDC-145: I. Expression on Dendritic Cells and Other Subsets of Mouse Leukocytes. Cell. Immunol..

[cit47] Flacher V., Tripp C. H., Stoitzner P., Haid B., Ebner S., Koch F., Park C. G., Steinman R. M., Idoyaga J., Romani N. (2010). Proteins deposited in the dermis are rapidly captured and presented by epidermal Langerhans cells. J. Invest. Dermatol..

[cit48] Cho H.-I., Barrios K., Lee Y.-R., Linowski A. K., Celis E. (2013). BiVax: a peptide/poly-IC subunit vaccine that mimics an acute infection elicits vast and effective anti-tumor CD8 T-cell responses. Cancer Immunol. Immunother..

[cit49] Hartmeier P. R., Ostrowski S. M., Busch E. E., Empey K. M., Meng W. S. (2024). Lymphatic distribution considerations for subunit vaccine design and development. Vaccine.

[cit50] Gall C. L., Cammarata A., de Haas L., Ramos-Tomillero I., Cuenca-Escalona J., Schouren K., Wijfjes Z., Becker A. M. D., Bödder J., Dölen Y., de Vries I. J. M., Figdor C. G., Flórez-Grau G., Verdoes M. (2022). Efficient targeting of NY-ESO-1 tumor antigen to human cDC1s by lymphotactin results in cross-presentation and antigen-specific T cell expansion. J. Immunother. Cancer.

[cit51] Liu M., Li J., Zhao D., Yan N., Zhang H., Liu M., Tang X., Hu Y., Ding J., Zhang N., Liu X., Deng Y., Song Y., Zhao X. (2022). Branched PEG-modification: A new strategy for nanocarriers to evade of the accelerated blood clearance phenomenon and enhance anti-tumor efficacy. Biomaterials.

[cit52] Harris T., Alabugin I. V. (2019). Strain and stereoelectronics in cycloalkyne click chemistry. Mendeleev Commun..

[cit53] Hering A., Emidio N. B., Muttenthaler M. (2022). Expanding the versatility and scope of the oxime ligation: rapid bioconjugation to disulfide-rich peptides. Chem. Commun..

[cit54] Pujari S. S., Zhang Y., Ji S., Distefano M. D., Tretyakova N. Y. (2018). Site-specific cross-linking of proteins to DNA via a new bioorthogonal approach employing oxime ligation. Chem. Commun..

[cit55] Kaur A., Baldwin J., Brar D., Salunke D. B., Petrovsky N. (2022). Toll-like receptor (TLR) agonists as a driving force behind next-generation vaccine adjuvants and cancer therapeutics. Curr. Opin. Chem. Biol..

[cit56] Salem M. L., Kadima A. N., Cole D. J., Gillanders W. E. (2005). Defining the Antigen-Specific T-Cell Response to Vaccination and Poly(I:C)/TLR3 Signaling: Evidence of Enhanced Primary and Memory CD8 T-Cell Responses and Antitumor Immunity. J. Immunother..

[cit57] Salem M. L., Diaz-Montero C. M., EL-Naggar S. A., Chen Y., Moussa O., Cole D. J. (2009). The TLR3 agonist poly(I:C) targets CD8+ T cells and augments their antigen-specific responses upon their adoptive transfer into naïve recipient mice. Vaccine.

[cit58] Zhang L., Dewan V., Yin H. (2017). Discovery of Small Molecules as Multi-Toll-like Receptor Agonists with Proinflammatory and Anticancer Activities. J. Med. Chem..

[cit59] Albin T. J., Tom J. K., Manna S., Gilkes A. P., Stetkevich S. A., Katz B. B., Supnet M., Felgner J., Jain A., Nakajima R., Jasinskas A., Zlotnik A., Pearlman E., Davies D. H., Felgner P. L., Burkhardt A. M., Esser-Kahn A. P. (2019). Linked Toll-Like Receptor Triagonists Stimulate Distinct, Combination-Dependent Innate Immune Responses. ACS Cent. Sci..

[cit60] Tom J. K., Albin T. J., Manna S., Moser B. A., Steinhardt R. C., Esser-Kahn A. P. (2019). Applications of Immunomodulatory Immune Synergies to Adjuvant Discovery and Vaccine Development. Trends Biotechnol..

[cit61] Gerlinger M., Rowan A. J., Horswell S., Larkin J., Endesfelder D., Gronroos E., Martinez P., Matthews N., Stewart A., Tarpey P., Varela I., Phillimore B., Begum S., McDonald N. Q., Butler A., Jones D., Raine K., Latimer C., Santos C. R., Nohadani M., Eklund A. C., Spencer-Dene B., Clark G., Pickering L., Stamp G., Gore M., Szallasi Z., Downward J., Futreal P. A., Swanton C. (2012). Intratumor Heterogeneity and Branched Evolution Revealed by Multiregion Sequencing. N. Engl. J. Med..

[cit62] Leko V., Rosenberg S. A. (2020). Identifying and Targeting Human Tumor Antigens for T Cell-Based Immunotherapy of Solid Tumors. Cancer Cell.

[cit63] Cattaneo C. M., Battaglia T., Urbanus J., Moravec Z., Voogd R., de Groot R., Hartemink K. J., Haanen J. B. A. G., Voest E. E., Schumacher T. N., Scheper W. (2023). Identification of patient-specific CD4+ and CD8+ T cell neoantigens through HLA-unbiased genetic screens. Nat. Biotechnol..

[cit64] Fennemann F. L., de Vries I. J. M., Figdor C. G., Verdoes M. (2019). Attacking Tumors From All Sides: Personalized Multiplex Vaccines to Tackle Intratumor Heterogeneity. Front. Immunol..

[cit65] Alspach E., Lussier D. M., Miceli A. P., Kizhvatov I., DuPage M., Luoma A. M., Meng W., Lichti C. F., Esaulova E., Vomund A. N., Runci D., Ward J. P., Gubin M. M., Medrano R. F. V., Arthur C. D., White J. M., Sheehan K. C. F., Chen A., Wucherpfennig K. W., Jacks T., Unanue E. R., Artyomov M. N., Schreiber R. D. (2019). MHC-II neoantigens shape tumour immunity and response to immunotherapy. Nature.

[cit66] Kreiter S., Vormehr M., van de Roemer N., Diken M., Löwer M., Diekmann J., Boegel S., Schrörs B., Vascotto F., Castle J. C., Tadmor A. D., Schoenberger S. P., Huber C., Türeci Ö., Sahin U. (2015). Mutant MHC class II epitopes drive therapeutic immune responses to cancer. Nature.

[cit67] Charles T., Moss D. L., Bhat P., Moore P. W., Kummer N. A., Bhattacharya A., Landry S. J., Mettu R. R. (2022). CD4+ T-Cell Epitope Prediction by Combined Analysis of Antigen Conformational Flexibility and Peptide-MHCII Binding Affinity. Biochemistry.

[cit68] Jensen K. K., Andreatta M., Marcatili P., Buus S., Greenbaum J. A., Yan Z., Sette A., Peters B., Nielsen M. (2018). Improved methods for predicting peptide binding affinity to MHC class II molecules. Immunology.

[cit69] Hos B. J., Tondini E., Camps M. G. M., Rademaker W., van den Bulk J., Ruano D., Janssen G. M. C., de Ru A. H., van den Elsen P. J., de Miranda N. F. C. C., van Veelen P. A., Ossendorp F. (2022). Cancer-specific T helper shared and neo-epitopes uncovered by expression of the MHC class II master regulator CIITA. Cell Rep..

