# Supplementary material for: Co-delivery of antigen and adjuvant by site-specific conjugation to dendritic cell-targeted Fab fragments potentiates T cell responses
Source: RSC Chem Biol. 2025 May 5;6(6):948–62. doi: 10.1039/d5cb00014a (PMC12057635; doi:10.1039/d5cb00014a)
Supplement: CB-006-D5CB00014A-s001 [file CB-006-D5CB00014A-s001.pdf]

Supplementary Information (SI) for RSC Chemical Biology.
This journal is © The Royal Society of Chemistry 2025

Supplemental information

Synthesis TLR7/8a-PEG$_{5k}$-DBCO:

General considerations:

DBCO-PEG$_5$-NHS was obtained from Click Chemistry Tools (Newark, USA) and 1-(4-(Aminomethyl)benzyl)-2-butyl-1H-imidazo[4,5-c]quinolin-4-amine dihydrochloride was obtained from MedChemExpress (Monmouth Junction, USA). Solvents were purchased from Merck Milipore (Darmstadt, Germany), and were used as received. Analytic LC/MS was performed using a hybrid Thermo Finnigan/Shimadzu system equipped with a Phenomenex Gemini NX C18 column (3 µm, 110 Å, 150 mmL x 2.0 mmD) using mobile phases A: 1% aq. HCOOH, B: 1% HCOOH in MeCN, 5-100% B/A gradient, 45 min with a flow of 0.2 mL/min with detection at 210 – 600 nm by a photo diode array (PDA), coupled to a LCQ fleet mass spectrometer (Thermo Finnigan ESI ion-trap) (LRMS). Peaks were manually integrated using MestreNova Software. High resolution mass spectrometry (HRMS) (ESI-TOF) was recorded on a AccuTOF CS JMS-T100CS mass spectrometer (JEOL), equipped with an electrospray ion source in positive mode (source voltage 2.4 kV, capillary temperature 250 °C) with resolution R = 7000 (mass range = 10 – 10 000). Preparative HPLC was performed using a Shimadzu system equipped with a Phenomenex Gemini NX-C18 column (5 µm, 110 Å, 150 mmL x 21.2 mmD) in combination with mobile phases A: 50 mM aq. NH_4HCO_3, B: MeCN, with detection at 215/254 nm. A gradient of 5-80% B/A was used, in 40 min with a flow of 6 mL/min. ^{1}H NMR and ^{13}C NMR spectra were recorded on a Bruker Avance III 500 (11.7 T) spectrometer. Chemical shifts (δ) are reported in ppm relative to tetramethylsilane as internal standard or the residual signal of the deuterated solvent was used as reference point. Coupling constants (J) are given in Hz. All ^{13}C APT experiments were proton decoupled.

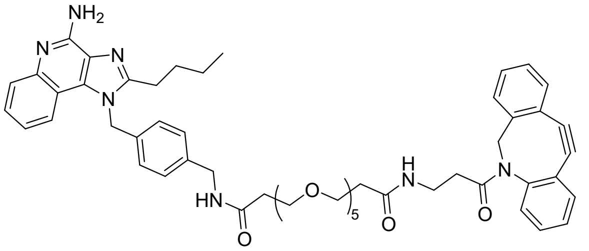

TLR-7/8a-PEG$_{5k}$-DBCO. A round-bottom flask was charged with 1-(4-(Aminomethyl)benzyl)-2-butyl-1H-imidazo[4,5-c]quinolin-4-amine dihydrochloride (9.1 mg, 20.95 µmol, 1 eq), equipped with a stirring bar and flushed with Ar. Dry DMF (500 µL) was added and the solution was stirred. Et_3N (7.3 µL, 52.38 µmol, 2.5 eq) was added to the stirring solution. Meanwhile, DBCO-PEG$_5$-NHS (12.7 mg, 18.31 µmol, 0.9 eq) was dissolved in dry DMF (500 µL) and the resulting solution was added to the stirring mixture. The mixture stirred at RT under Ar overnight, after which it was diluted with 50 mM NH_4HCO_3 aq. sol. (1 mL) and MeOH (1 mL). The mixture was purified by reversed phase preparative basic HPLC (Rt: 29 min). Lyophilization resulted in the title compound as a colourless oil (6.05 mg, 6.44 µmol, 31%). ^{1}H NMR (500 MHz, DMSO) δ 8.29 (t, *J* = 6.0 Hz, 1H), 7.77 (d, *J* = 8.2 Hz, 1H), 7.67 (t, *J* = 5.7 Hz, 1H), 7.62 (d, *J* = 7.4 Hz, 1H), 7.57 (td, *J* = 6.7, 2.9 Hz, 2H), 7.52 – 7.42 (m, 3H), 7.42 – 7.27 (m, 5H), 7.18 (d, *J* = 7.9 Hz, 3H), 7.03 (t, *J* = 7.3 Hz, 1H), 6.96 (d, *J* = 7.9 Hz, 3H), 6.56 (s, 2H), 5.83 (s, 3H), 5.03 (d, *J* = 14.1 Hz, 1H), 4.21 (d, *J* = 5.9 Hz, 3H), 3.67 – 3.55 (m, 4H), 3.52 – 3.37 (m, 15H), 3.15 – 3.05 (m, 1H), 2.97 – 2.84 (m, 4H), 2.33 (t, *J* = 6.3 Hz, 3H), 2.16 (t, *J* = 6.5 Hz, 2H), 1.80 (ddd, *J* = 15.9, 8.4, 5.7 Hz, 1H), 1.70 (p, *J* = 7.6 Hz, 3H), 1.37 (h, *J* = 7.4 Hz, 3H), 1.23 (s, 1H), 0.86 (t, *J* = 7.4 Hz, 3H). ^{13}C NMR (126 MHz, DMSO) δ 170.61, 170.34, 139.21, 135.46, 130.01, 128.68, 128.52, 128.19, 128.06, 127.27, 126.87, 125.86, 125.67, 122.92, 120.55, 70.19, 70.11, 69.96, 69.90, 67.29, 67.13, 55.29, 42.00, 36.57, 36.40, 35.40, 34.63, 30.02, 26.68, 22.31, 14.16. HPLC: Rt. 18.62 min. UV purity: >95%. LRMS (ESI+) *m/z* calc. for $C_{54}H_{64}N_7O_8$ $[M+H]^+$ = 938.47, found 938.74. HRMS (ESI+) *m/z* calc. for $C_{54}H_{64}N_7O_8$ $[M+H]^+$ = 938.4811, found 938.4792.

Supplementary figures

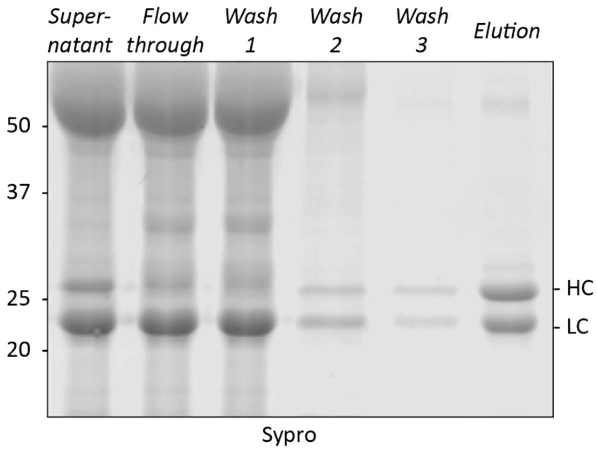

Supplemental figure 1| Ni-NTA Isolation anti-DEC205 Fab-srt-His. The anti-DEC205 Fab fragment equipped with sortase-recognition motif and His-tag was isolated in high yield (70 mg Fab fragment) using Ni-NTA-His tag affinity isolation. The purity was determined by reducing 12% SDS-PAGE analysis and was determined to be >95%.

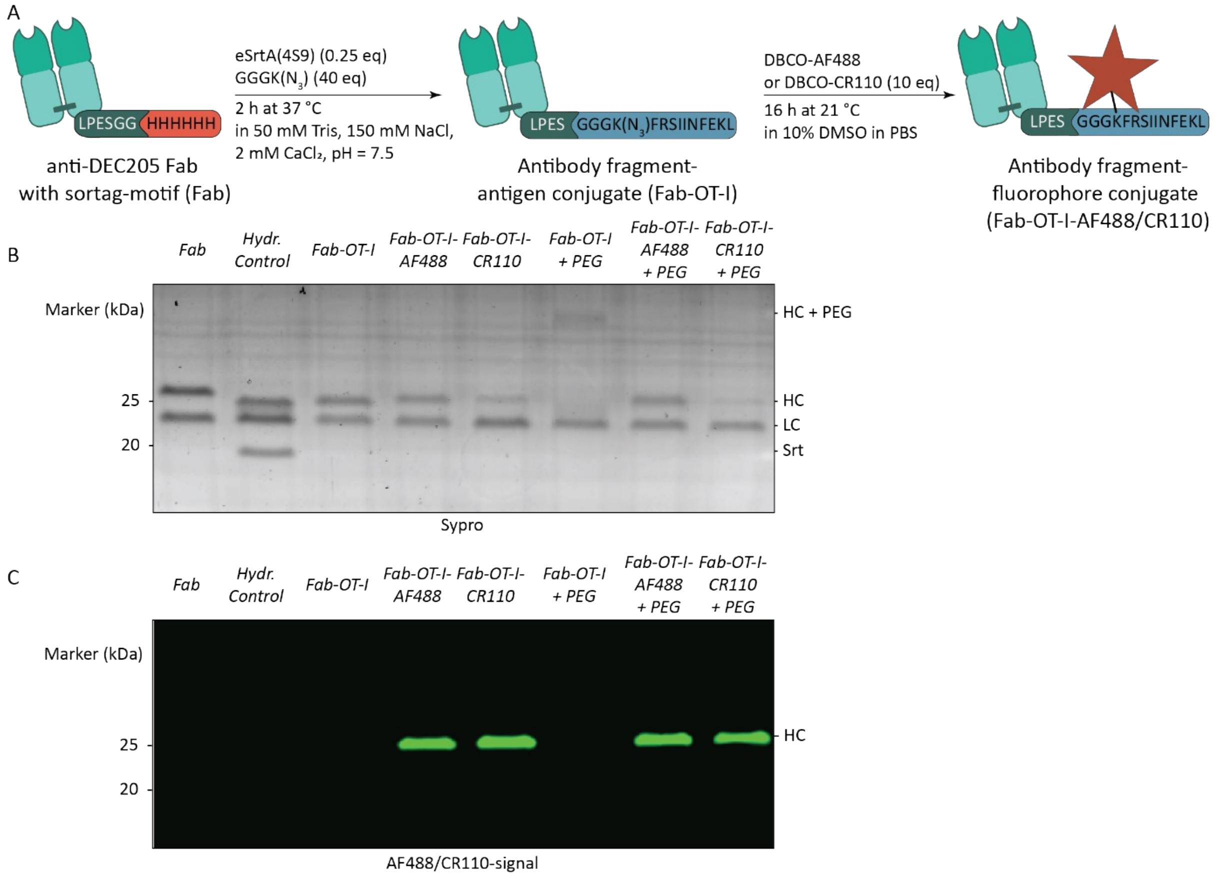

Supplemental figure 2| Characterization of fluorescently labeled Fab-antigen conjugates. A) Schematic overview of reaction conditions to obtain AlexaFluor-488 and carboxyrhodamine-110 labeled conjugates (Fab-OT-I-AF488/CR110). B) Reducing SDS-PAGE (12%) analysis of Fab-OT-I-AF488 and Fab-OT-I-CR110. Analytical click reaction with DBCO-PEG$_{5k}$ demonstrates availability azide in lane 6 by a mass shift corresponding to attachment of the PEG$_{5k}$, whereas in lane 7 and lane 8 no shift is observed as the azide is consumed by the DBCO-adjuvant. C) Fluorescent reducing SDS-PAGE (12%) analysis of Fab-OT-I-AF488 and Fab-OT-I-CR110. A fluorescent signal is observed on the heavy chains of the Fab fragments at the expected molecular height of 25 kDa, indicating product formation.

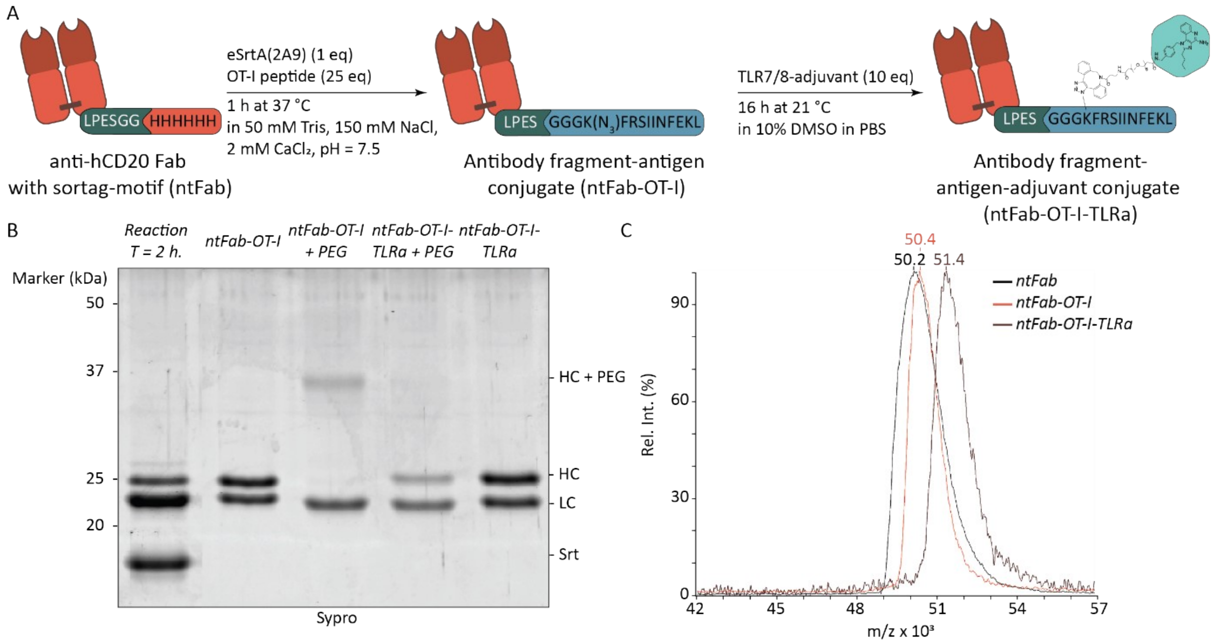

Supplemental figure 3|Characterization of non-targeted (anti-hCD20) AAA-conjugate. A) Schematic overview of reaction conditions to obtain AAA-conjugate (ntFab-OT-I-TLRa). B) Reducing SDS-PAGE (12%) analysis of ntFab-OT-I-TLRa. Analytical click reaction with DBCO-PEG$_{5k}$ demonstrates availability azide in lane 3 by a mass shift corresponding to attachment of the PEG$_{5k}$, whereas in lane 5 no shift is observed as the azide is consumed by the DBCO-adjuvant. C) MALDI-TOF analysis of Fab fragments. Mass shifts correspond to removal of His-tag and attachment of antigen and adjuvant respectively.

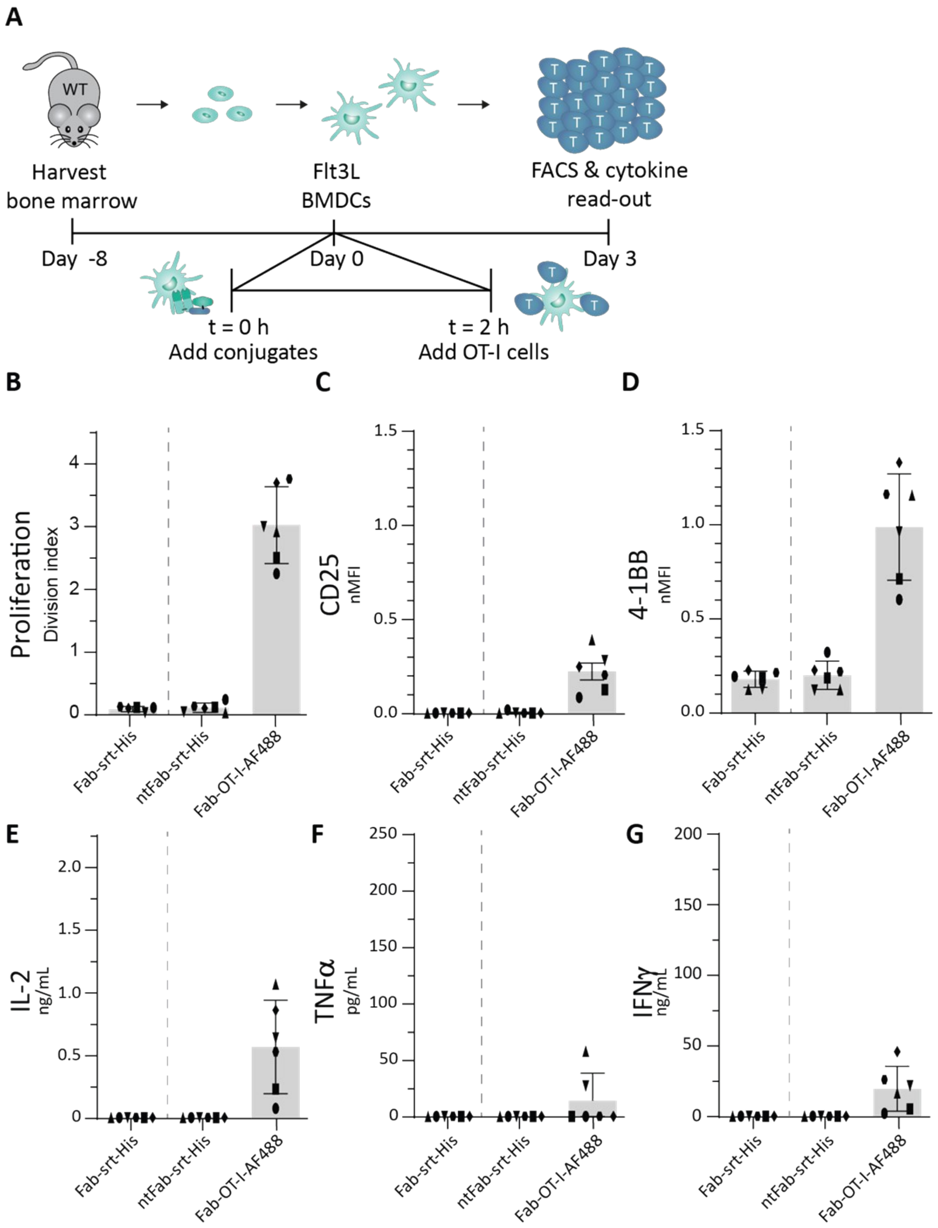

Supplemental figure 4| *Ex vivo* analysis of Fab-srt-His, ntFab-srt-His and Fab-OT-I-AF488. A) Schematic overview of *ex vivo* T cell activation assay. In short, Flt3L-BMDCs were generated and pulsed for 2 h with 10 nM conjugate. Sequentially, the BMDCs were washed and a co-culture of BMDCs and OT-I cells (1:5 ratio) was set-up. After 3 days, OT-I cells were analyzed using FACS and cytokines in the supernatant were assessed via ELISA. B-D) Flow cytometry analysis of OT-I cells. Data (n = 6, technical duplicates) are depicted as division index (B) and as mean fluorescence intensity normalized to positive control ±SD for CD25 (C) and 4-1BB (D). E-G) ELISA analysis (n = 6, technical duplicates) of IL-2 (E), TNFα (F), and IFNγ (G). Data is depicted as mean ±SD.

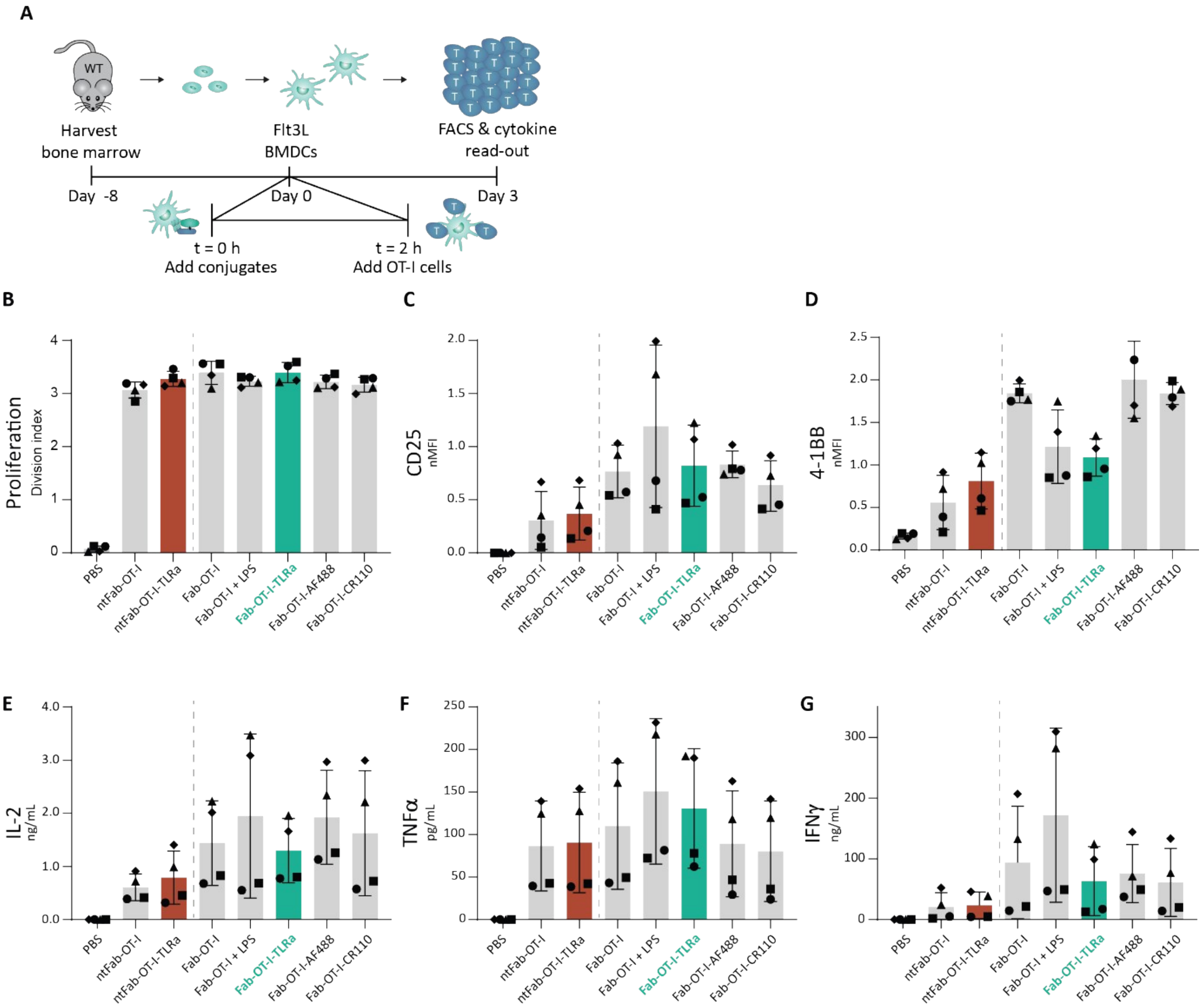

Supplemental figure 5| *Ex vivo* analysis of AAA-conjugates at higher concentration (100 nM) masks beneficial effects of antigen-adjuvant co-delivery. A) Schematic overview of *ex vivo* T cell activation assay. In short, Flt3L-BMDCs were generated and pulsed for 2 h with 100 nM conjugate. Sequentially, the BMDCs were washed and a co-culture of BMDCs and OT-I cells (1:5 ratio) was set-up. After 3 days, OT-I cells were analyzed using FACS and cytokines in the supernatant were assessed via ELISA. B-D) Flow cytometry analysis of OT-I cells. Data (n = 4, technical duplicates) are depicted as division index (B) and as mean fluorescence intensity normalized to positive control ±SD for CD25 (C) and 4-1BB (D). E-G) ELISA analysis (n = 4, technical duplicates) of IL-2 (E), TNFα (F), and IFNγ (G). Data is depicted as mean ±SD.

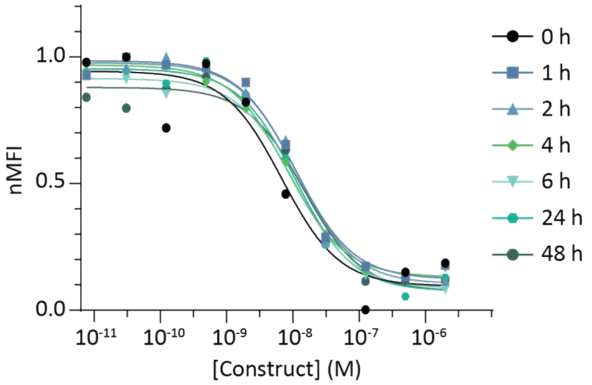

Supplemental figure 6| Serum stability of AAA-conjugate. The AAA-conjugate was incubated in 50% mouse serum in PBS for up to 48 h at 37 °C. Afterwards, a competitive binding assay using DEC205-expressing JAWS II cells was performed and no indication of majorly reduced binding capacity was observed for the conjugates incubated in serum.

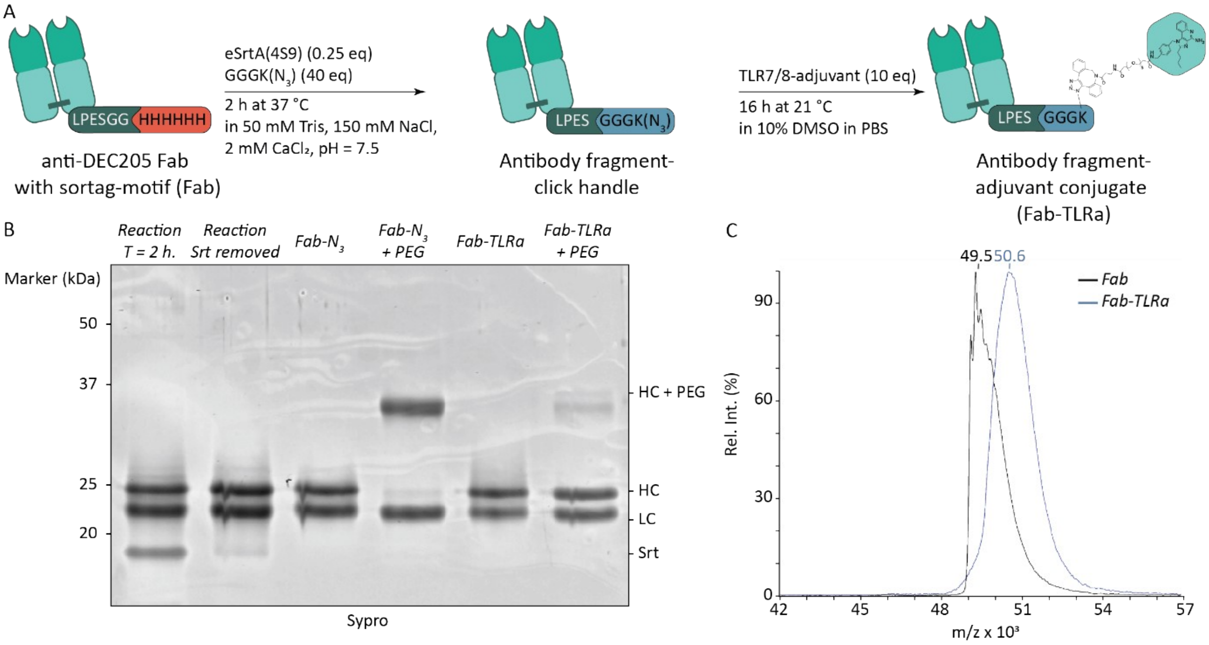

Supplemental figure 7|Characterization of targeted adjuvant conjugate. A) Schematic overview of reaction conditions to obtain targeted adjuvant conjugate (Fab-TLRa). B) Reducing SDS-PAGE (12%) analysis of Fab-TLRa. Analytical click reaction with DBCO-PEG$_{5k}$ demonstrates availability azide in lane 4 by a mass shift corresponding to attachment of the PEG$_{5k}$, whereas in lane 6 no shift (<5%) is observed as the azide is consumed by the DBCO-adjuvant. C) MALDI-TOF analysis of Fab fragments. Mass shifts correspond to removal of His-tag and attachment of adjuvant.

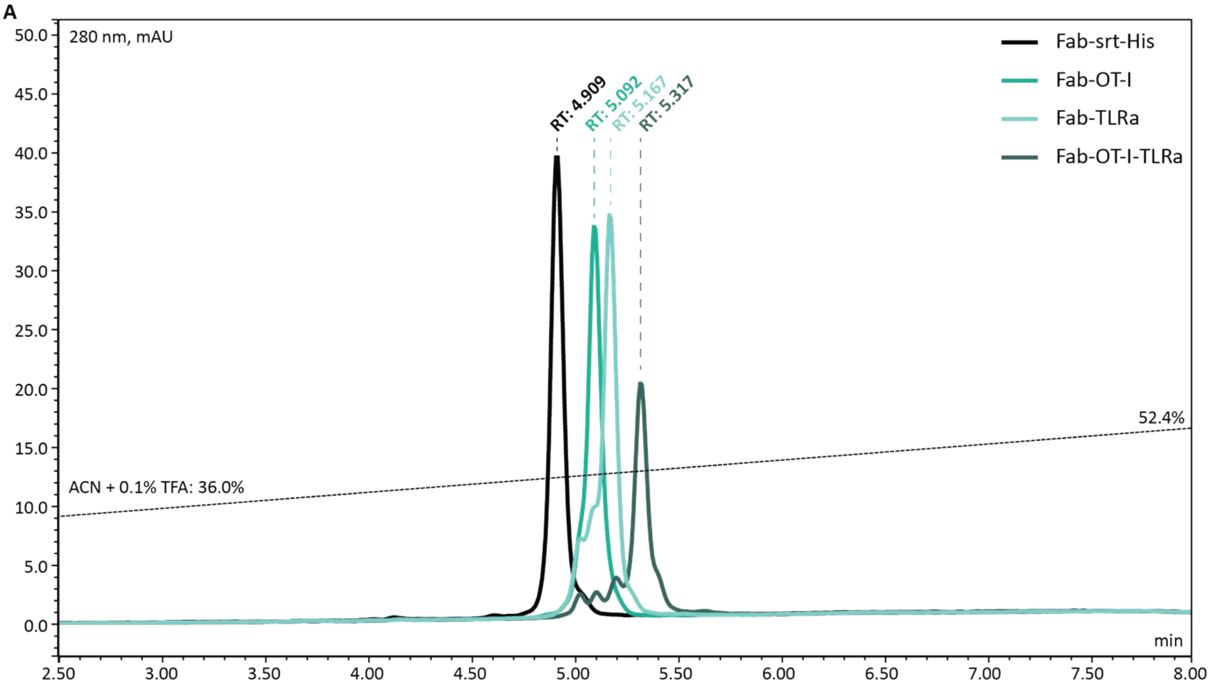

Supplemental figure 8|RP-HPLC analysis of conjugates. A) RP-HPLC analysis (280 nm) of Fab-srt-His (retention time = 4.909 min), Fab-OT-I (retention time = 5.092 min), Fab-TLRa (retention time = 5.167 min), and Fab-OT-I-TLRa (retention time = 5.317 min). The dotted line depicts the gradient used during the analysis.

Supplemental tables

Supplemental Table 1|Sequence of 4S9 HDR-template

5’ Homology arm	CCTGGAACTCTGGAGCCCTGTCCAGCGGTGTGCACACCTTCCCAGCTGTCCTGCAGTCTGGAC<br>TCTACACTCTCACCAGCTCAGTGACTGTACCCTCCAGCACCTGGTCCAGCCAGGCCGTCACCT<br>GCAACGTAGCCCACCCGGCCAGCAGCACCAAGGTGGACAAGAAAATTGGTGAGAGAACAAC<br>CAGGGGATGAGGGGCTCACTAGAGGTGAGGATAAGGCATTAGATTGCCTACACCAACCAGG<br>GTGGGCAGACATCACCAGGGAGGGGGCCTCAGCCCAGGAGACCAAAAATTCTCCTTTGTCTC<br>CCTTCTGGAGATTTCTATGTCCTTTACACCCATTTATTAATATTCTGGGTAAGATGCCCTTGCA<br>TCATGACATACAGAGGCAGACTAGAGTATCAACCTGCAAAAGGTCATACCCAGGAAGAGCCT<br>GCCATGATCCCACACCAGAACCAACCTGGGGCCTTCTCACCTATAGACCATACTAACACACAG<br>CCTTCTCTCTGCA
Hinge region + GGGS-linker + Sortag + Histag	GTGCCAAGGGAATGCGGAGGCGGAGGCAGCCTGCCGGAATCCGGCGGCCACCATCACCATC<br>ACCATTGA
Internal ribosome entry site (IRES)	GGATCCCAATTGCTCGAGGCCCCTCTCCCTCCCCCCCCCCTAACGTTACTGGCCGAAGCCGCT<br>TGGAATAAGGCCGGTGTGCGTTTGTCTATATGTTATTTTCCACCATATTGCCGTCTTTTGGCAA<br>TGTGAGGGCCCGGAAACCTGGCCCTGTCTTCTTGACGAGCATTCCTAGGGGTCTTTCCCCTCT<br>CGCCAAAGGAATGCAAGGTCTGTTGAATGTCGTGAAGGAAGCAGTTCCTCTGGAAGCTTCTT<br>GAAGACAAACAACGTCTGTAGCGACCCTTTGCAGGCAGCGGAACCCCCCACCTGGCGACAG<br>GTGCCTCTGCGGCCAAAAGCCACGTGTATAAGATACACCTGCAAAGGCGGCACAACCCCAGT<br>GCCACGTTGTGAGTTGGATAGTTGTGGAAAGAGTCAAATGGCTCTCCTCAAGCGTATTCAAC<br>AAGGGGCTGAAGGATGCCCAGAAGGTACCCCATTGTATGGGATCTGATCTGGGGCCTCGGT<br>GCACATGCTTTACATGTGTTTAGTCGAGGTTAAAAAACGTCTAGGCCCCCCGAACCACGGGG<br>ACGTGGTTTTCCTTTGAAAAACACGATGATAATATGGCCACAGAATTCGCCACC
blasticidin resistance	ATGGCCAAGCCTTTGTCTCAAGAAGAATCCACCCTCATTGAAAGAGCAACGGCTACAATCAA<br>CAGCATCCCCATCTCTGAAGACTACAGCGTCGCCAGCGCAGCTCTCTCTAGCGACGGCCGCA<br>TCTTCACTGGTGTCAATGTATATCATTTTACTGGGGGACCTTGTGCAGAACTCGTGGTGCTGG<br>GCACTGCTGCTGCTGCGGCAGCTGGCAACCTGACTTGTATCGTCGCGATCGGAAATGAGAAC<br>AGGGGCATCTTGAGCCCCTGCGGACGGTGCCGACAGGTGCTTCTCGATCTGCATCCTGGGAT<br>CAAAGCCATAGTGAAGGACAGTGATGGACAGCCGACGGCAGTTGGGATTCGTGAATTGCTG<br>CCCTCTGGTTATGTGTGGGAGGGCTAAG
Poly(A)-tail	TACTAGTCGACTGTGCCTTCTAGTTGCCAGCCATCTGTTGTTTGCCCCTCCCCCGTGCCTTCCT<br>TGACCCTGGAAGGTGCCACTCCCACTGTCCTTTCCTAATAAAATGAGGAAATTGCATCGCATT<br>GTCTGAGTAGGTGTCATTCTATTCTGGGGGGTGGGGTGGGGCAGGACAGCAAGGGGGAGG<br>ATTGGGAAGACAATAGCAGGCATGCTGGGGATGCGGTGGGCTCTATGGAGATCTTTAATTA<br>AGGT
3’ Homology arm	AAGTCACTAGGACTATTACTCCAGCCCCAGATTCAAAAAATATCCTCAGAGGCCCATGTTAGA<br>GGATGACACAGCTATTGACCTATTTCTACCTTTCTTCTTCATCTACAGGCTCAGAAGTATCATC<br>TGTCTTCATCTTCCCCCCAAAGACCAAAGATGTGCTCACCATCACTCTGACTCCTAAGGTCACG<br>TGTGTTGTGGTAGACATTAGCCAGAATGATCCCGAGGTCCGGTTCAGCTGGTTTATAGATGA<br>CGTGGAAGTCCACACAGCTCAGACTCATGCCCCGGAGAAGCAGTCCAACAGCACTTTACGCT<br>CAGTCAGTGAACTCCCCATCGTGCACCGGGACTGGCTCAATGGCAAGACGTTCAAATGCAAA<br>GTCAACAGTGGAGCATTCCCTGCCCCCATCGAGAAAAGCATCTCCAAACCCGAAGGTGGGA<br>GCAGCAGGGTGTGTGGTGTAGAAGCTGCAGTAGGCCATAGACAGAGCTTGACTTAACTAGA<br>CTT

Supplemental table 2| Endotoxin results

Conjugate	*Endotoxin levels (EU/mL)*
Fab	<0.01
Fab-OT-I	<0.02
Fab-OT-I-TLRa	<0.02
Fab-OT-I-AF488	<0.02
Fab-OT-I-CR110	<0.03
ntFab	<0.01
ntFab-OT-I	<0.01
ntFab-OT-I-TLRa	<0.01
Fab-TLRa	<0.01
ntFab-TLRa	<0.01
